# Beyond Numbers: Determining the Socioeconomic and Livelihood Impacts of African Swine Fever and Its Control in the Philippines

**DOI:** 10.3389/fvets.2021.734236

**Published:** 2022-02-10

**Authors:** Tarni L. Cooper, Dominic Smith, Mark Jaypee C. Gonzales, Marlon T. Maghanay, Sunny Sanderson, Marie Rachelle Jane C. Cornejo, Lohreihleih L. Pineda, Rose Ann A. Sagun, Oliver P. Salvacion

**Affiliations:** ^1^School of Agriculture and Food Sciences, The University of Queensland, Brisbane, QLD, Australia; ^2^School of Veterinary Science, The University of Queensland, Brisbane, QLD, Australia; ^3^Griffith Asia Institute, Griffith University, Brisbane, QLD, Australia; ^4^College of Veterinary Medicine, Central Bicol State University of Agriculture, Pili, Philippines; ^5^College of Veterinary Science and Medicine, Central Luzon State University, City of Munoz, Philippines

**Keywords:** African Swine Fever, participatory research, Philippines, value chain, livelihoods, smallholders, socioeconomics

## Abstract

The impacts of African Swine Fever (ASF) have most frequently been described quantitatively though it is increasingly acknowledged these impacts extend well beyond numbers. During 2020, a multidisciplinary team of researchers developed a framework for Socioeconomic and Livelihood Impact Assessment (SELIA) of livestock diseases in smallholder communities. Two key innovations within this SELIA framework are the integration of sustainable livelihoods concepts to capture rich information beyond financial impacts, and the inclusion of stakeholders across the value chain, beyond farmers. This paper focuses on the findings from one of the first applications of the SELIA framework. In late 2020 the research team applied participatory tools from the SELIA Framework (8 focus group discussions, 14 key informant interviews, and 2 network mapping activities) to gather data to describe the impact of ASF in backyard pig-farming communities and value chains. This was undertaken across two locations in the Philippines, in turn highlighting potential leverage points for intervention. Owing to COVID-19 travel restrictions and risks, modifications to training and field activities were made. Findings from focus groups and interviews revealed the deep, emotional impacts of ASF and the associated control measures. Pigs were considered pets by many farmers and some women described them as being like their children. Animal health-workers (AHWs) also recognised the emotional toll on farmers and were sometimes strongly criticised by community members due to their involvement in depopulation campaigns. Misinformation early in the epidemic also led farmers to hide their animals from AHWs, and to dispose of them inappropriately. While the overall impact of ASF on society was negative, the impacts across different communities, scales of production and different value chain actors varied. The losses experienced by backyard farmers resulted in significant losses to linked value chain actors, such as input suppliers. This trial application of the SELIA framework revealed some complex and varied impacts of ASF. This included significant differences in livelihood and socio-economic impacts amongst different actors within value chains and also among different categories of actors (for example small, medium and large-scale traders). Repeated themes and triangulated findings suggest two leverage points for further consideration. Firstly, it is recommended a One Welfare approach to ASF control in the Philippines is explored. Emphasising careful communication between animal health-workers and farmers, and humane and sensitive pig depopulation practices. Secondly, consideration of ASF support programs tailored to sectoral and specific communities is recommended.

## Introduction

**Figure 1:**
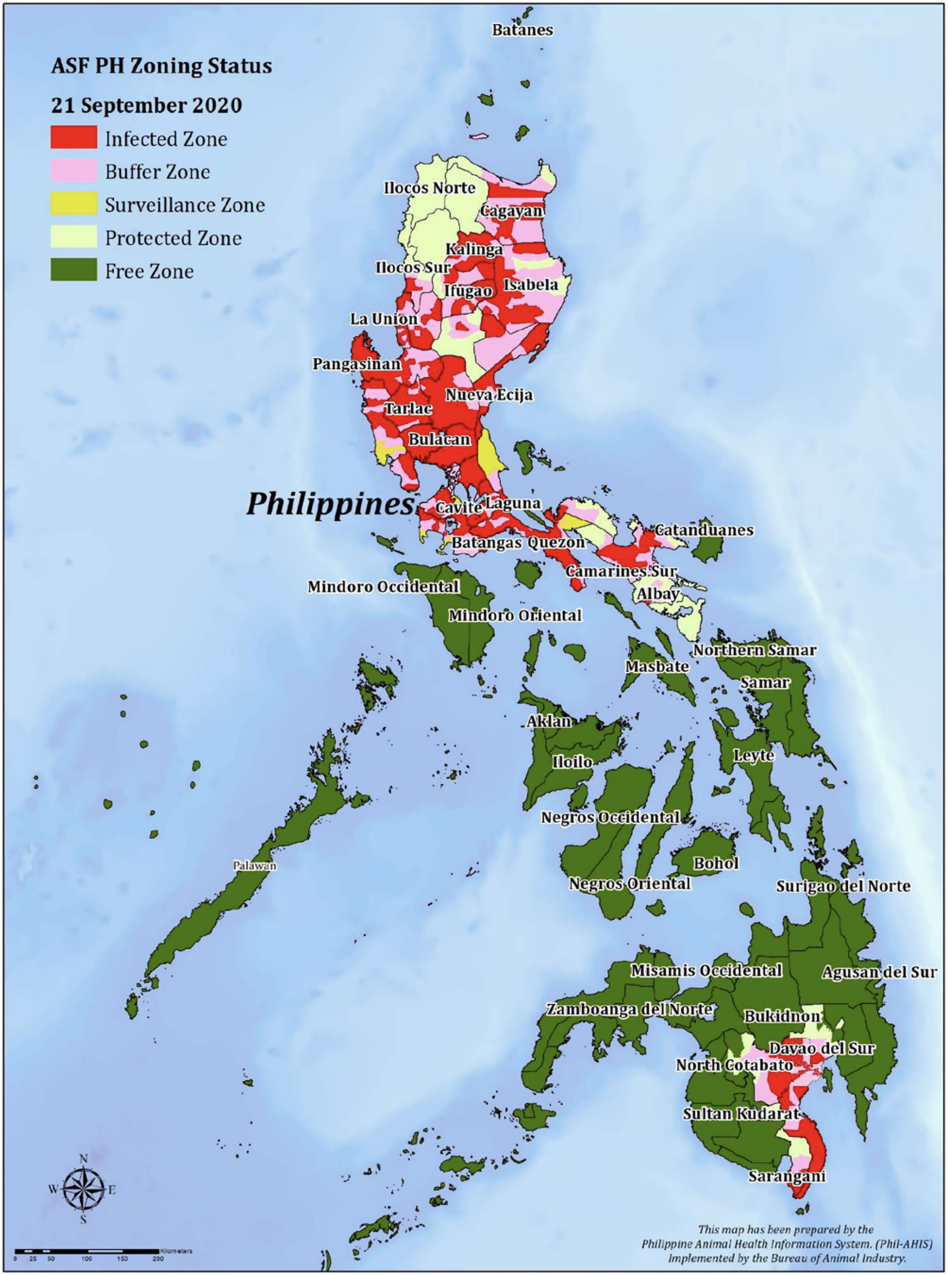
ASF zoning status in Philippines, September 2020.

African Swine Fever (ASF) was first reported in the Philippines in July 2019, starting with seven outbreaks in the province of Rizal, Region IV-A, adjacent to Metro Manila (National Capital Region) in the Philippines (1). As of 21st September 2020, ASF had been reported in 31 provinces across eight regions. A further nine provinces where ASF was not reported by the 21st September 2020 were classified as buffer, surveillance or protected zones (2) (Figure 1). The outbreak of ASF resulted in a 9.8% drop in pig production in the last quarter of 2019 (3). The Government of the Philippines continued to collect quantitative data throughout the outbreak for both larger-scale commercial holdings and smaller farms (2). What have been less-well understood are the broader impacts of ASF on tangible and intangible, qualitative aspects of livelihoods, both within farming households and the broader value chains. There has been expressed a need by governments and international non-government organisations for this sort of information across Southeast Asia and the Pacific (1).

**Figure 2:**
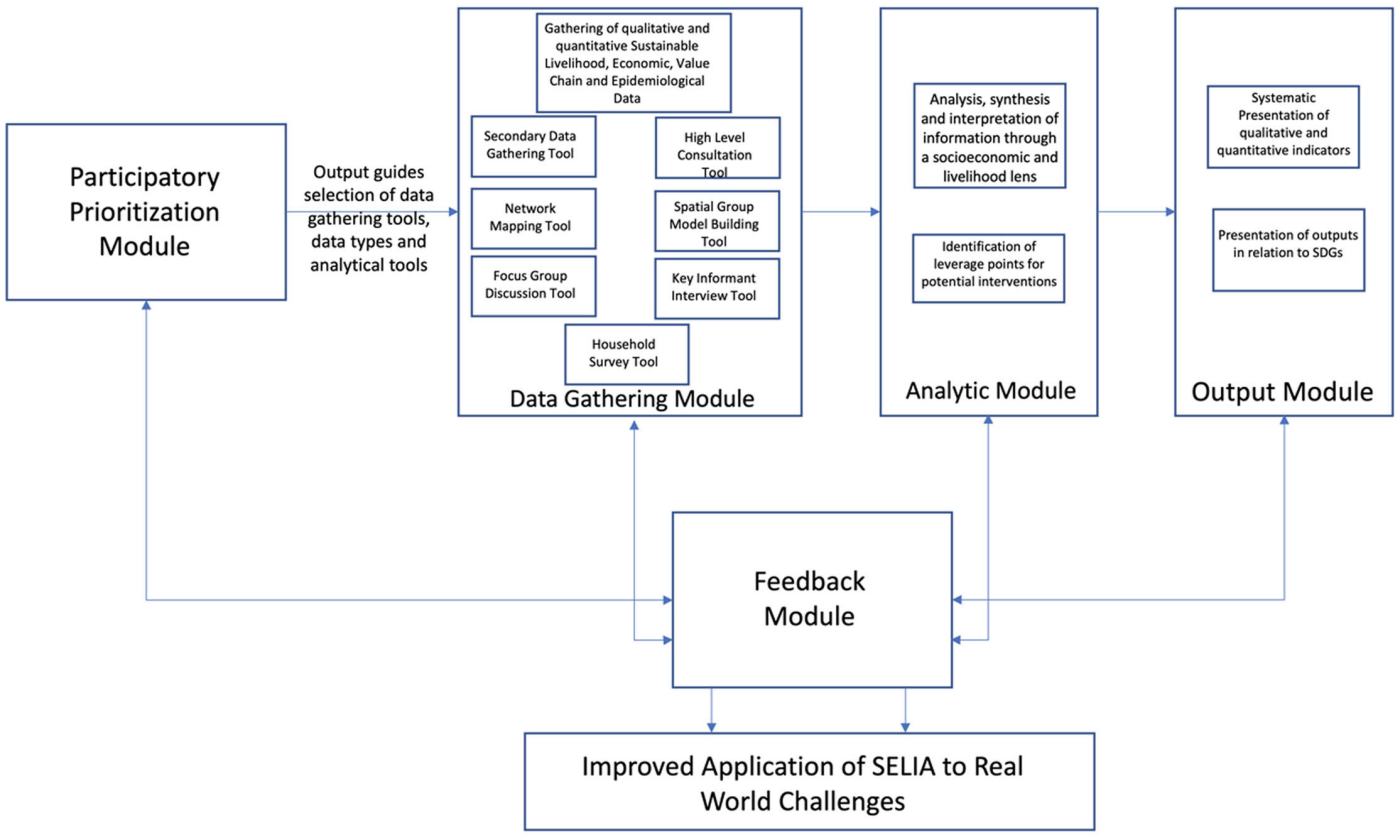
Schematic representation of the Socioeconomic and Livelihood Impact Assessment (SELIA) Framework for livestock diseases.

In response, from early 2020, university and government partners in Australia, Timor-Leste and the Philippines developed a Socioeconomic and Livelihood Impact Assessment (SELIA) Framework for livestock diseases (Figure 2) with an initial focus on ASF (4). The SELIA Framework is modular in design with both an overarching process of participatory prioritisation with decision-makers, such as government or donors, followed by data gathering, analysis and creation of useful outputs. In addition to this linear process there is a continual feedback component, where the researchers use formative evaluation to improve the assessment process in dialogue with the decision-makers. The SELIA Framework is designed to be adaptable to different decision-maker priorities, research needs and resource availabilities, and so each assessment is likely to look very different. The SELIA Framework's first trial applications have been this study and another in Timor-Leste during late 2020 (5).

The term “livelihood” is interpreted in many ways, from a synonym for “income,” to a mixed tangible and intangible phenomenon. This has led to a multiplicity of livelihood-associated frameworks, methods and tools. In the SELIA context, livelihood impact assessment is based around the Sustainable Livelihoods Framework (SLF) as proposed by the Department for International Development (DFID) (6) and widely adopted since the 1990s. The SLF includes many components of a livelihood for interrogation, such as the vulnerability context in which a livelihood is set, livelihoods capitals (social, human, natural, physical, and financial) used to create this livelihood and transforming structures and processes influencing the way these capitals can be utilised, such as laws, policies, culture, institutions and gender dimensions. The SLF is further based on the key principals that any interventions must be participatory and responsive; multi-level; conducted in partnership with the private and public sector; sustainable and dynamic (7). The SELIA framework may have future applications in framing discussion, planning of interventions, implementation and monitoring and therefore, and could hold utility in examining how communities might use their resources to address livestock biosecurity threats.

The risks posed by livestock diseases such as ASF extend well beyond production losses (8). Agricultural diseases often have significant indirect and sometimes direct impacts on human health and wellbeing, both tangible and intangible, with ripple effects through communities. The speed and severity of the ASF epidemic in the pig sectors of Southeast Asia and the Pacific Region has left stakeholders scrambling to determine appropriate management and/or control measures. ASF has been a shock to pig raising systems that has resulted in both tangible and intangible impacts due to the personal and cultural significance of pigs in many regions. Further, communities, households and individuals have different vulnerability to the impact of ASF, and as such, an understanding of relative vulnerability is critical for effective decision-making in at-risk countries. The original motivation for the development of the SELIA Framework was to evaluate the impacts of livestock disease on the livelihoods of those often most vulnerable, smallholder farmers. The Framework was soon expanded to include impacts along the value chains, as the impacts of ASF extend well beyond the farmgate.

Value chain analysis takes a systems approach to analysis and enables an understanding of the overall market system and context. Taking a systemic approach allows for any challenges, problems, and bottlenecks at various points within the value chain to be identified (9). Taking a systemic approach is very important for impact assessment frameworks like SELIA, as this enables the identification of flow-on impacts of shocks to producers across different levels of the value chain.

**Figure 3:**
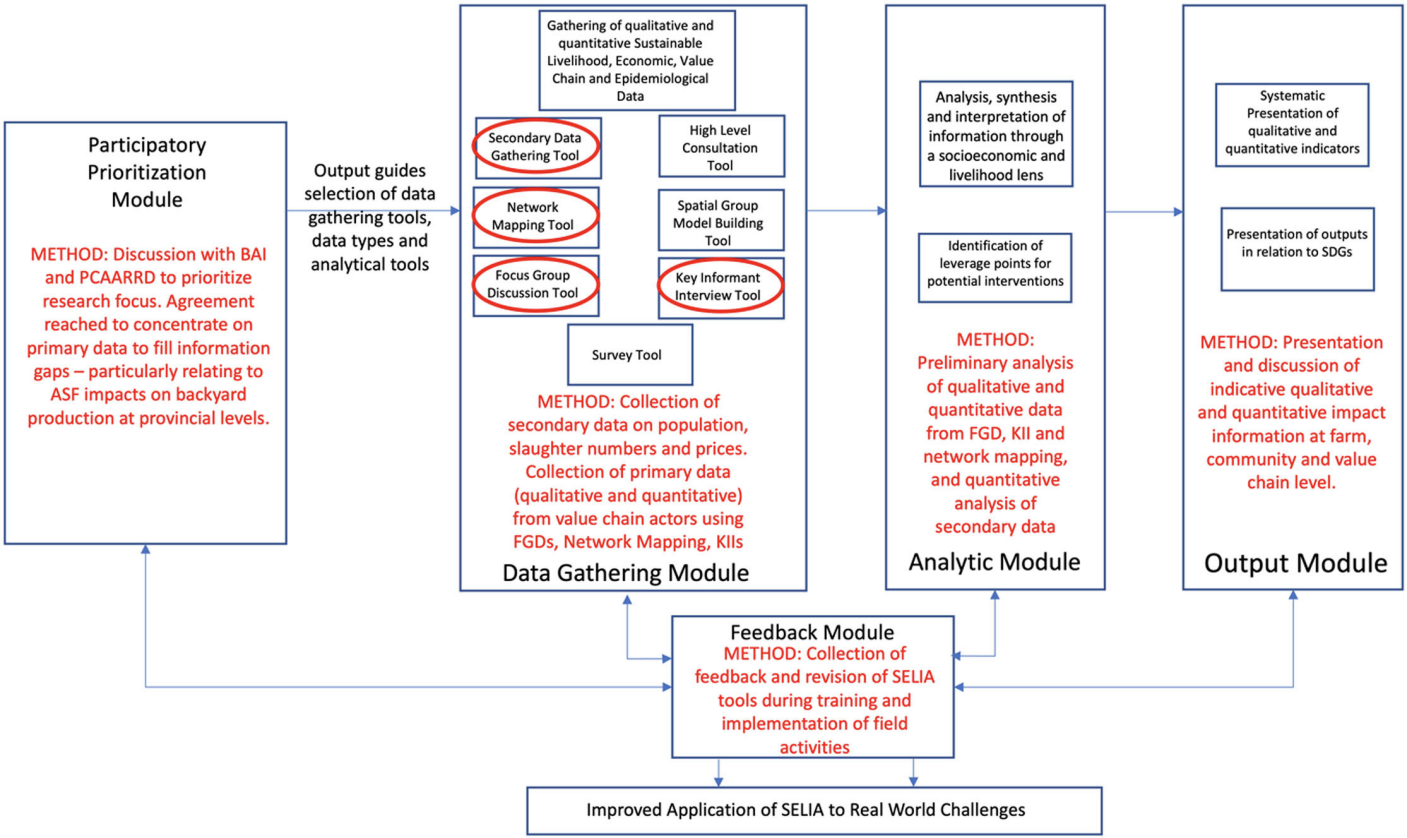
Pilot implementation of the SELIA Framework in the Philippines (selected tools and applications in red).

This paper describes the trial application of a set of core data gathering tools from the SELIA Framework in two locations in the Philippines, to better understand the impact of ASF in smaller-scale pig-pork value chains (Figure 3).

## Materials and Methods

### The Philippines Country Context and Site Selection

The Philippines is an archipelago in Southeast Asia that has a population of 106.7 million people (10), over half of whom live in rural areas (52.85%) (11). In 2019, approximately one in five of the total population lived in poverty (20.8%) (12). Furthermore, from 2017 to 2019, on average 17.6% of the population suffered severe food insecurity (13). Agriculture is of crucial importance in the Philippines, and closely linked to food security and poverty reduction (14). As the country was already grappling with the large-scale outbreak of ASF in 2020, the impact of COVID-19 delivered an additional blow to livelihoods and poverty reduction efforts in the Philippines.

Philippine partners for this research included the Department of Agriculture's Bureau of Animal Industry (BAI), Department of Science and Technology's Philippine Council for Agriculture, Aquatic, and Natural Resources Research and Development (PCAARRD), Central Luzon State University (CLSU) and Central Bicol State University of Agriculture (CBSUA). Two sites were chosen for field research with one key inclusion criterion, that the communities studied had been impacted by ASF. Other than meeting this criterion, sites were chosen to mitigate COVID-19 risk by reducing travel for researchers. The two locations chosen were Camarines Sur province, Bicol Region and Nueva Ecija province, Central Luzon Region, focusing on San Jose City.

Camarines Sur has 35 municipalities and two cities. The province's first ASF outbreak was in Barangay Sto. Domingo, Bombon on February 21, 2020. The situation quickly spread to nearby municipalities such as Canaman, Calabanga, Magarao and Naga City (Bicol's Centre of Commerce and Industry). The ASF situation in Camarines Sur is still evolving; as at 18th of August 2020 ASF was reported in 17 municipalities and one city^1^, but as of September 21, ASF was active in 18 municipalities and both cities.

In San Jose City, Nueva Ecija, ASF first emerged in Barangay Santo Nino on January 3, 2020, and since, there have been a total of 18 outbreaks in San Jose City^2^ In Nueva Ecija province, 29 of the 32 cities and municipalities are infected with ASF (red zones) and three remain as buffer zones.

### Prioritisation and Instruments Tested

Firstly, the research team held discussions with BAI and PCAARRD to establish research needs and priorities. National and regional level data to base these rapid estimations on were readily available online from the Philippine Statistics Authority (PSA) (https://psa.gov.ph/pages/survey). However, government partners explained it had been difficult to assess the full impacts and extent of ASF due to the additional resources required to gather detailed information at the local level, and the impact of COVID-19 on resourcing and movement restrictions. Analysis of secondary data confirmed these gaps in information. Details of the secondary data analysis are included in the Supplementary Materials.

Given the gaps in knowledge in how ASF was affecting smallholders, their communities and connected value chain actors, and owing to resource, time and COVID-safety constraints for the pilot activities, the following primary data collection methods were chosen: Focus Group Discussions (FGDs) (with farmers), Network Mapping (with mixed value chain actors) and Key Informant Interviews (KIIs). Details of COVID-19 safe measures adopted for these data collection methods are given in section COVID-19 Risk Mitigation. Details of the instruments used to guide these methods are included as attachments and described briefly below. A total of eight FGDs, two Network Mapping sessions and 14 KIIs were conducted across the two sites (see Table 1).

**Table 1:** Activities conducted and details of participants in each study location.

**Activity**	**Nueva Ecija, Central Luzon**	**Camarines Sur, Bicol**
Key informant interviews	1. Livestock Inspector at City Veterinary Office (male) 2. Animal handler and ASF response team at City Veterinary Office (female) 3. Agri-supply business owner (female) 4. Agri-supply business owner (female) 5. City Veterinarian, meat inspector and regulator (male) 6. City Slaughterhouse Master (female) 7. Pig trader (male) 8. Pig trader/meat vendor (female)	1. Meat Inspector at City Veterinary Office/Animal Health Worker (female) 2. Pig trader and pork seller (male) 3. Meat inspector at locally registered meat establishment (male) 4. Team Leader (feed monitoring) at a private company 5. Senior Meat Control Officer (male) 6. Piggery utility worker, backyard pig raiser, butcher, private livestock technician (male)
Focus Group Discussions	1. Female part time pig farmers, women, (average age 50yo) 2. Male part-time pig farmers, men (average age 53yo) 3. Female full time (commercial) pig farmers, women (average age 55yo) 4. Male full time (commercial) pig farmers, men (average age 41yo)	1. Female pig farmers with 10 or fewer pigs, 5 women, (average age 45yo) 2. Male pig farmers with 10 or fewer pigs, 5 men (average age 45yo) 3. Female pig farmers with >10 pigs, 6 women (average age 50yo) 4. Male pig farmers with >10 pigs, 7 men (average age 57yo)
Network Mapping	One group of participants (9 men, 7 women, average age 45yo): i. Veterinary Officer/Animal Health Worker x 2 ii. Housewife/pig farmer x 4 iii. Farmer/pig farmer x 7 iv. Call centre agent/pig farmer x 1 v. Poultry supply owner/agri-input supplier x 1 iv. Meat stall owner/pig trader x 1	One group of participants (4 men, 1 woman, average age 26yo): i. Meat Inspector at City Veterinary Office/Animal Health Worker ii. Self-employed, feed retailer, pig farmer iii. LGU veterinarian iv. Animal technician/livestock inspector LU v. Student, son of pig farmer

#### Network Mapping

Mapping the value chain processes and key actors and product flows gives an overview of the key stakeholders within a sector (15), enabling decisions about the bounds of the analysis, and which actors are most usefully included within an impact assessment (16), i.e., it informs subsequent research design and sampling. In this study, a qualitative and semi-quantitative value chain mapping exercise concentrated on product flow, coordination, governance and linkages aspects. Mapping techniques were utilised to build up an accurate picture of actor types, numbers of actors, flow volumes, values, prices, costs and benefits and the participation of the poor. Governance and linkages were incorporated into the analysis with a concentration on analysing social capital and coordination and cooperation inside and outside the value chains based on the inclusive value chain analysis methodology outlined in (9). The mapping process is outlined in detail in the Supplementary Material.

The mapping served to build up a picture of the key categories of actors, the volume of flow of products between actors, the value of products at each level of the chain, the costs and benefits to different actors, the number of actors at each level of the chain and the linkages between the actors. This provided the foundation for an in-depth and contextually embedded initial estimation of impacts on upstream and downstream actors of ASF at producer level in terms of value and volumes of product.

#### Focus Group Discussions

Focus Group Discussions were designed to gather contextual, community-level data on the impacts of ASF on pig farmers. Participatory activities included community timelines, seasonal calendars, and collection of epidemiological information through proportional piling, ranking, tabulation and open discussion. Participatory epidemiological methods policies (17, 18) are more sensitive than surveys for capturing local, contextual information and hold great utility for rapid assessments of disease impact in the field. To understand the vulnerability context of the community, seasonal calendars were developed to study disease and risk factors, population structures, disease features, biosecurity, disease timeline—historical data, disease impacts, strategies employed, plans for the future, indicative farm budget information, and responses to disease at community level.

Gathering farmers together for discussion aimed to first explore the role of pigs in the livelihoods of communities in the context of whole, usually very complex livelihoods. In SELIA, the FGD guide includes all elements of the SLF, either explicitly or in the discussion probed by the facilitator. This gives the researchers an understanding of the underlying vulnerability context and potential resilience of the community to the livestock disease. Following on from this, the FGD zooms in on this disease to better understand how the disease has and is impacting the livelihoods of the farming community. The FGD finishes with a discussion of the livelihood strategies the farmers are employing and plan to employ in the future, to mitigate the impacts of the disease.

#### Key Informant Interviews

Semi-structured, key informant interviews are usually the main method for primary data collection of actors beyond the farm. Semi-structured interviews are not based on a rigid sequence of short and precise quantitative questions as is the case with structured interviews. Instead, they consist of a series of exchanges and discussions around pre-determined questions and topics following a flexible interviewing format.

Targets for KIIs were identified through high-level consultations, review of secondary data and the FGD and network mapping exercises, and with assistance from the Local Government Units in the study sites. Two broad categories of key informants were targeted:

- Direct or indirect market participants (*n* = 5 in Central Luzon and *n* = 3 in Central Bicol): These are either involved in the marketing, and processing of the agricultural commodity under analysis (e.g., traders and processing firms) or engaged in the delivery of commercial services to value chain participants (e.g., input suppliers and transporters). These value chain actors are able to give detailed information about prices, costs, flows and linkages between actors and narratives of personal impacts. In particular, KIIs with direct value chain participants concentrated on: (i) the respondent's role in the value chain; (ii) the characteristics of purchasing products; (iii) characteristics of product selling; (iv) understanding costs and profits; (v) impact of animal disease on the business of the interviewee; (vi) opinion of impact of animal disease on other value chain actors; and (vii) hopes for the future.
- Knowledgeable observers (*n* = 3 in Central Luzon and *n* = 3 in Central Bicol): These people do not participate in the production and marketing of the commodity in question but may offer important information and insights. In general, academics, researchers, retired food industry managers, policy makers, other government officials, extension officers, and staff from donor agencies, NGOs, or projects all fall under this category. The knowledgeable observers targeted in this study were limited to those associated with animal health. Animal Health Workers (AHWs) can give a rich description of disease context and impacts on a population-health level. The checklist topics for these interviews included: (i) the respondent's role in animal health; (ii) fees for services; (iii) disease timeline; (iv) strengths/successes in disease response; (v) weaknesses/challenges in disease response; (vi) disease impact on farmers and other value chain actors; (vii) disease impact on themselves; and (viii) hopes for the future.

### Training and Roles of Field Researchers

Field researchers were trained by the lead institution using a combination of interactive Zoom sessions, practice activities, and trainer and peer feedback. Three training sessions of 3-3.5 h were structured around PowerPoint presentations, which were also provided in advance of the sessions for printing. The first session was dedicated to Key Informant Interviews and Foundational Principles, such as the overview and aims of the project, roles and responsibilities and research ethics. The remaining two sessions covered Focus Group Discussions and Network Mapping, and were mostly hands-on. The outputs from the two field teams for every activity were reviewed by the training team who provided feedback both during Zoom sessions and in between each session. Following the sessions, university teams practiced using the tools and provided further practice outputs for review.

The team size was kept to a minimum (three people) for COVID-19 risk mitigation. The three major roles for each team were a lead facilitator, a note-taker and an observer, with the latter two supporting the lead facilitator as needed. The note-taker plays a very important role as they capture discussions that would not be captured by the other activity outputs. As the research was a pilot, the observer role was responsible for observing what worked and didn't work during the research process and suggesting improvements. The team gathered after each activity to reflect on the process and supplement notes taken. The team later transcribed and translated all materials with one person taking the lead and the others checking the outputs.

### Materials

The field teams were required to source materials for training and field research. The materials were basic, cheap and readily available to the teams: blank A0 paper (“flipchart paper”), markers, coloured paper, legumes, a measuring jug and paper for note taking. To reduce sharing of pens for hygiene reasons, the teams also bought a stack of post-it notes for each participant to write on and stick on the shared A0 paper. For COVID-19 risk mitigation during face-to-face activities, materials included face masks, face shields, liquid soap, gloves, hand sanitiser, alcohol with foot dispenser, foot bath with rugs for drying, a temperature scanner, a health declaration logbook and health declaration check list. Refreshments for these meetings included snacks (biscuits, bread, spaghetti, *palabok, pancit*, and beverages) and meals (prepared by a local person or fast food).

### Human Research Ethics

The research proposal and tools underwent review and were approved by The University of Queensland Human Research Ethics Committee (approval #2020001543).

### Participant Recruitment

The teams were responsible for seeking consent from gatekeepers at the Local Government Unit (LGU) level before approaching and seeking consent from prospective participants. The teams translated research information sheets and provided these to gatekeepers and participants before seeking free and informed consent.

Sampling for Key Informant Interviews was purposive; the interviewees were selected to ensure each of the four stakeholder-specific interview guides could be piloted. Sampling for Network Mapping was also purposive, aiming to bring together voices across the pig-pork value chain.

For FGDs, the aim was to include two different scales of smallholder enterprise. In each location, the proposed FGD group classifications were slightly different; in Nueva Ecija, researchers defined smaller scale as “part-time” pig raisers (where income streams are heavily mixed) and slightly larger as “full-time” pig raisers. In Camarines Sur the research team used the number of pigs kept as the defining feature as they explained, it is common even for larger scale farmers to have mixed livelihoods. The research team divided participants on whether they owned 10 or fewer pigs, or 11 or more as provided by the Municipal Agriculture Office of Pamplona, which was based on their latest list and depopulation report.

The total number of participants were 33 in Camarines Sur and 39 in Nueva Ecija. The details of these are listed in Table 1.

### Data Analysis

Once all primary data from KIIs and participatory group activities (FGDs, Network Mapping) were collected, a thematic analysis was used to analyse interview transcripts, field notes and other participatory group activity outputs. Initially, a deductive approach was taken, using sustainable livelihood themes based on the SLF and associated codes. In addition, as additional themes emerged, inductive coding was used, adding additional codes to the pre-determined list. Relationships between codes and themes were then identified and the findings were interpreted within the wider context of the research. The findings were discussed and agreed upon by the research team and reported to government partners for their feedback. Primary quantitative data underwent descriptive analysis.

### COVID-19 Risk Mitigation

No researchers travelled internationally, and all training and collaboration occurred online. Six field researchers from two remote institutions were trained online and worked closely with the local Departments of Health (DoH) to ensure the research was implemented safely. Precautions included screening of field researchers for COVID-19, the use of personal protective equipment and social distancing. Where there was a greater prevalence of COVID-19 in the community, activities were conducted online or via telephone.

## Results

The results below are organised according to the major socioeconomic and livelihood themes identified. They include both the ways in which livelihoods characteristics augment the impacts of ASF and the ways ASF impact upon livelihood characteristics. Findings are drawn from all field notes, transcripts and other research activity outputs.

### Vulnerability Context

**Figure 4:**
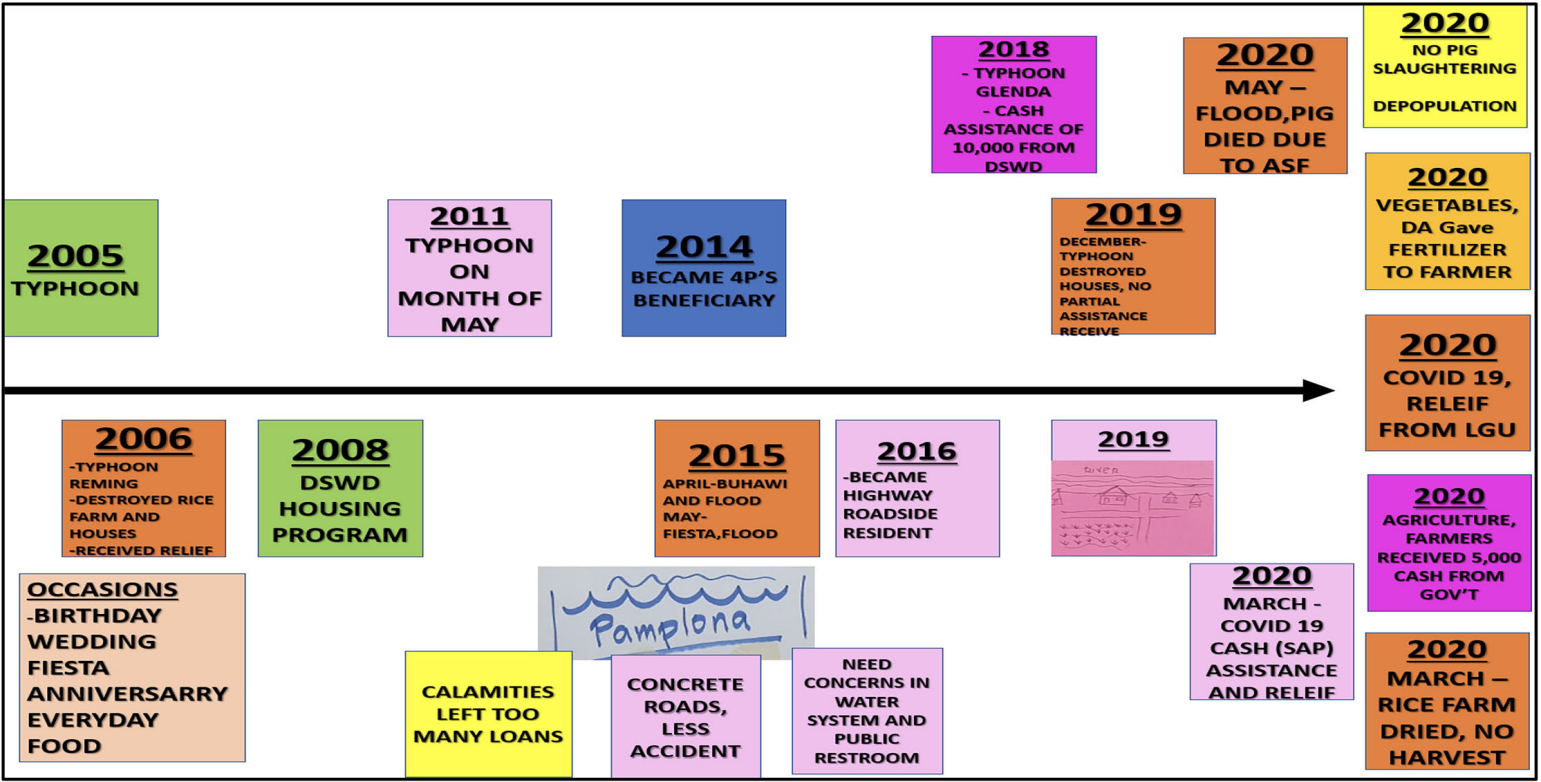
Community timeline developed by women farmers during focus group discussion—CSFGD1.

Communities differed in their underlying vulnerability to livelihood stressors. In Camarines Sur, an overriding vulnerability and cause of perpetual community anxiety was typhoons, occurring with seasonal regularity. As revealed in the FGD community timelines (Figure 4) and described by the note-taker:

*The participants started with talking about typhoons, which stimulated discussion of fear. One always feared for her children. Another exclaimed that typhoons would always cause floods, and she fears for the pigs during floods – CSFGD3 women*.

**Figure 5:**
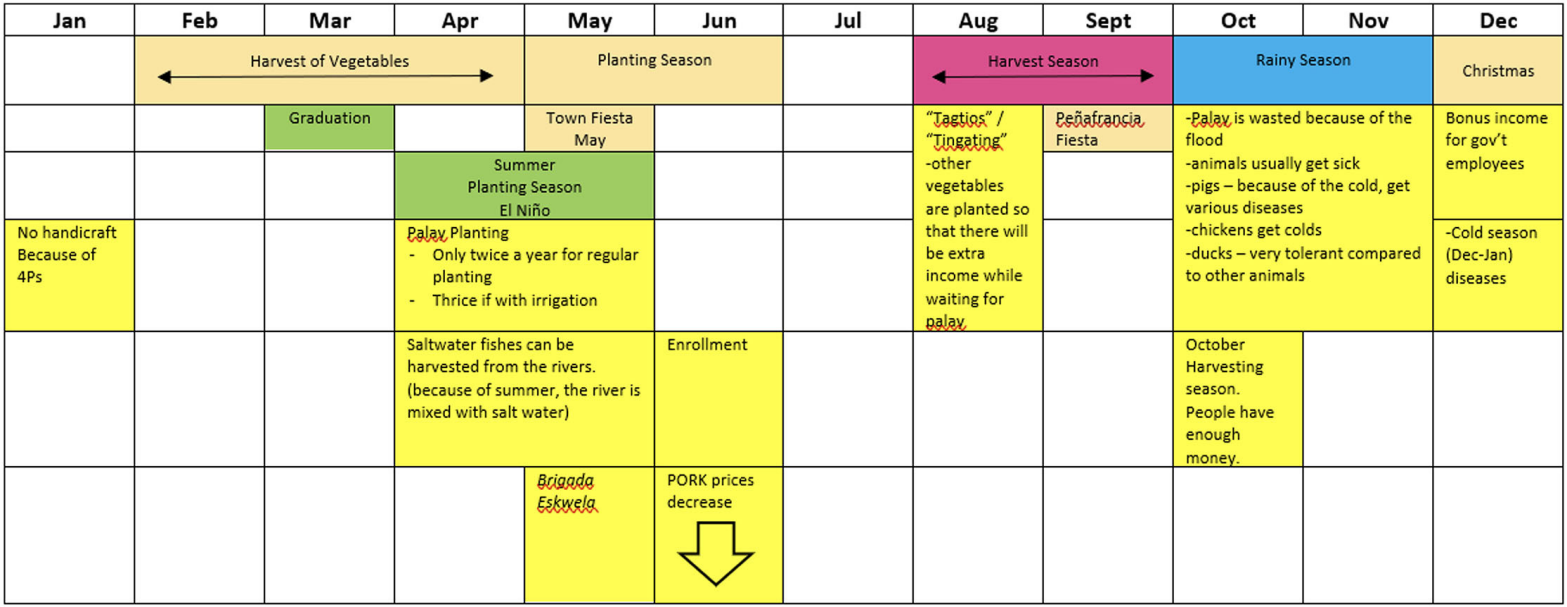
Seasonal calendar constructed by male participants during a focus group discussion in Camarines Sur—Male farmers of 10 pigs or fewer CSFGD2.

The seasonal calendar activity in FGDs provided information on how livelihood vulnerabilities change over a year. As well as weather events and income-generating activities, cultural events may result in seasonal variation in income. In Camarines Sur, Graduation Month (in March, at the end of the school year) brings slaughter and consumption of beef (for wealthier households) and pork (Figure 5). Losses to ASF during this period are particularly profound.

In August, participants in all Camarines Sur FGDs mentioned the word *Tingating* to describe it as a period of financial difficulty. There was also a seasonal component to common pig diseases described by the communities.

*August is a season of hardships. This is also usually the planting season which means no income for the farmers. To be able to get money for their daily expenses, the males in the family usually work as construction workers. The males also help in making nipas (traditional huts) and carrying them. The females make the tiklad (nipa thatch). Other than nipa making, the females also apply for jobs as nannies and salesladies in Naga City. Other females also sell different things such as vegetables or put up sari-sari stores and karenderia (eatery serving mostly Filipino dishes) – (CSFGD1 women)*.

Also contributing to vulnerability context, farmers faced additional, ongoing challenges in pig production including high feed prices, no or limited informal credits provided by the input suppliers, low live weight prices from traders, disease, and high vaccine prices.

### Social Capital

Relationships between animal health workers and farmers were important for communicating ASF risk mitigation messages.

*The strength in the Animal Health Worker response was communication. The ASF outbreak in San Jose City was contained immediately because they communicated personally to the pig raisers from commercial to backyard farms – Interview with Animal Health Worker in Nueva Ecija*.

In Camarines Sur, animal health workers said they visited farms daily and covered all farms for which they were responsible every week. These relationships between animal health workers and farmers were credited with the advances in ASF control. The relationships were, however, put under significant strain during depopulation campaigns (see psychosocial impacts below).

**Figure 6:**
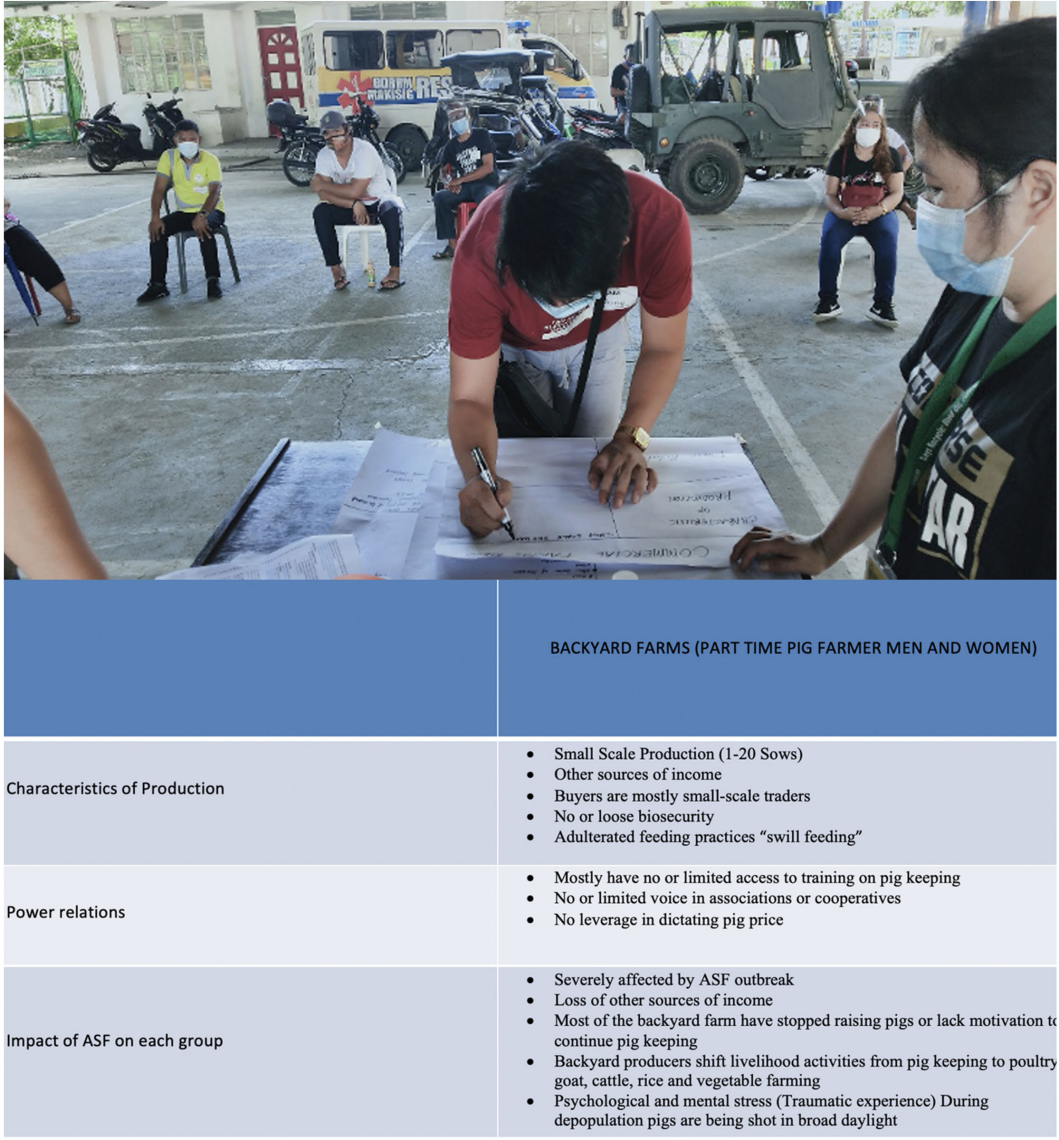
Farmers create a social inclusion matrix during a network mapping exercise in Nueva Ecija (photo top and digitised extract bottom).

During the Network Mapping activity, social inclusion mapping of the value chain was undertaken to highlight the heterogeneous nature of actors at various stages of the value chain and to explore the differing characteristics of production, power relations and the differential impact of disease between different groups within a value chain actor category (Figure 6).

No farmer networks or groups were mentioned by farmers. However, the collaborative nature of social inclusion matrix development also served to stimulate discussions about potential further collaboration and support between various actors in the value chain in order to respond to the challenges of ASF:

*They also discussed the need for organized pig farmer's associations or cooperatives in which they can enjoy privileges such as access to discounted inputs and have a voice in crafting political policy in their sector that the LGU will pass. NE 002 also added in their conversation, an organized barangay ASF response team [is needed] comprised of volunteer pig farmers, who are responsible for information dissemination and surveillance in their community. The social inclusion mapping for the participants became an eye-opener to them to unite together for one common goal of helping one another, for their sector to survive – NENM*.

### Financial Capital

Farmer livelihoods were complex, consisting of on-farm and off-farm activities and remittances. Proportional piling in the FGD placed pig farming within the context of an overall livelihood, hinting at the potential impact of pig production losses. While this was only intended to be a semi-quantitative, descriptive exercise, the triangulation of this activity with male and female groups repeatedly supported its accuracy; for example, males and females raising more than 10 pigs in Camarines Sur each said pigs made up 21% of their income. In Nueva Ecija, farmers described their province as “the rice granary of the Philippines” but pig farming was their second-most important income-generating activity. Male, commercial pig farmers said they contributed 30 percent, and this income was used for the education of their children and other necessities.

When reflecting on the pig production budgets they had made together in the FGDs, farmers described the impacts of ASF, including difficulties repaying debts:

*According to NE 031 the impact of ASF in each production budget line is really devastating to her, she cites with about the feeds that she has now an informal credit with his input supplier and very problematic on paying it. NE 032 discussed the effect of ASF in the price of the live weight of pigs which she stated to be on the range of 50 to 70 Php [USD1-USD1.40]/kg which really affected her income. Most member of FGDS discussed how ASF make their pig raising livelihood broke and had a negative income. And also, the pigs that died and buried which contribute to their loss income (NEFGD1)*.

Similar stories were shared during production of the process matrix in the Network Mapping activity. One participant had taken a loan from the bank to start his pig farm with his house serving as collateral and he could no longer service the loan.

Though the number of pigs on each smallholder farm was small, the losses to the sector overall and therefore to the associated value chains, were large. The losses were felt acutely by input suppliers and this gave them a vested interest in supporting farmers in biosecurity efforts:

*A lot of backyard farms were gone because of biosecurity, he said. He emphasised that the totality of all these small backyard pig raisers had a bigger impact on his targets than the larger farms. Thus, the lack of consumers caused their sales volumes to plummet. He also said, with a sigh, that some areas are only able to sell 20,000 bags in the month (instead of the usual 50,000)” – Interview with input supplier (CS004)*.

Network mapping allowed some quantification of losses across the value chain and comparison between production levels. Findings were triangulated with KIIs. Larger-scale farms tended to have a financial buffer to absorb some of the shock from ASF. Also, it became apparent that different value chain actors were impacted to varying degrees and some pig traders even benefited from ASF in the short term, taking advantage of panic selling of healthy pigs:

“*In terms of financial, she did not have losses during the ASF issue, instead her sales increased. Of course, she is not thankful that ASF hit their city that affected many especially the backyard farmers, but during those times, her business boomed… Her sales increased by at least 50% and that lasted for 3 months” – Interview with Pig trader (NE-008)*.

*In his opinion, the backyard farmers lost their livelihood and had credit from their feed supplier that up until now, they cannot repay. In that case, it was a domino effect on the feed supplier because pig raisers were not able to pay their debt. Then they had losses and were probably considered as bad debtors. Lastly, for traders like him, they won in this battle because some traders took the opportunity to have high sales – NE-007*.

FGD participants were not the only people with mixed livelihoods. All KII respondents had mixed livelihoods. No fees were charged for the government's veterinary services, they received a wage. However, animal health workers also had mixed livelihood activities. All were involved in the government ASF response, but one was also a pig farmer and two were engaged in some informal veterinary work on the side of their government jobs. One had a side business of artificial insemination providing him with around 8000Php (USD160) per month and another conducted pet vaccination and emergency care for about 2000Php (USD40).

Input suppliers also maintained side businesses as technicians which were impacted by ASF:

*Company technicians, to increase their sales volume and suki, would [usually] also act as livestock technicians. He mentions that it is very difficult today to utilize this side job because there are no backyard farms. Larger farms, on the other hand, do not need livestock technicians, he says, since these farms employ veterinarians – Input supplier (CS-004)*.

The fees charged for slaughtering within government establishments was inexpensive before and since ASF. In the example of small-scale slaughterhouses, the hot meat (informal, unregistered) butcher estimated he spent Php1200 on equipment and his rate for slaughtering was Php300/head. An LRME meat inspector described how they only charged Php105/head:

*The interviewers commented that it was cheap, to which CS-003 agrees and states, this is the reason why most people in Pamplona prefer their service than backyard slaughterers. He says that hiring a backyard slaughterer would cost Php300 (sometimes double, if you need two of them), and you have to consider them as guests. You offer food, cigarettes, and sometimes alcoholic drinks. He also mentions that this [the LRME] price did not change even after ASF and even they would get compliments that it is cheap (he would even joke to them, “Would you like us to increase cost?”) – Researcher notes on interview with meat inspector (CS-003)*.

While government slaughterhouse workers were largely protected from financial ASF impacts, one respondent noted that some butcher assistants, *saluyot*'s lost their jobs. Researchers interviewing the hot meat butcher noted the sadness in his eyes when he said that during 2020, he did not have a single customer for butchering or for his other business as a technician.

### Human Capital and Psychosocial Impacts

Smallholder farmers used the sale of pigs for important expenses such as education and ASF compromised this. As well as this more tangible impact of ASF, an emergent theme was the psychosocial impacts of ASF. Findings from focus groups and interviews revealed the deep, emotional impacts of ASF and associated control measures.

Open discussion with farmers revealed the intangible significance of pigs in their lives:

“*Pigs are like (our) children. (We) would often talk with them and would even cry when they are being sold. Even the youngest considers their pigs as family members” – Female participant, CSFGD3*.

The researchers described the tears of participants as they talked about the toll of ASF; they frequently spoke of the trauma of watching or hearing their pigs being shot under the depopulation effort. The note-taker in Nueva Ecija described varying degrees of “emotional shock,” “stress,” “depression,” and “sadness” across all groups:

*A participant recounted that she was crying while her pigs and piglets were starting to be culled during the depopulation because she witnessed from afar how the gunshot sounds pierced her ears and watched her pigs die simultaneously. She was in shock knowing that the proceeds from the sale of her pigs were intended for the schooling of her family, University Students in CLSU. She also recounted how she became heartbroken to see how her pigs suffered and was in shock and distress knowing that she would be economically on the brink due to an informal credit she got from her ‘Suking Agri Supply' (their input supplier trusted partner) – Female part-time pig farmer, 62yo (NE032, NEFGD1)*.

*The participant NE 011 recounted that he was furious at first while his pigs and piglets were starting to be depopulated because from afar, the gunshots pierced his ears. He was in shock knowing that the proceeds from the sale of his pigs, intended as his household income was already gone… You can see in the eyes of the participants how badly they were affected by the outbreak and how hard for them to bury their pigs seeing [the devastation] with their own eyes… - Male part-time pig farmers (NEFGD2)*.

*A participant shared her experiences when the City veterinary office depopulated their pigs. She cried and begged, “Ako na lang sana ang idamay nyo wag na ang aking mga alaga” (Please don't hurt my animals, hurt me instead). When depopulating she did not even look at her pigs. Instead, she went to other places to breathe. In addition, their investment [in the pigs should have helped] them to pay for their debt and additional income too. The owner was in turmoil physically and mentally especially when they remembered their everyday routine in working at their piggery, feeding, bathing, giving vitamins to their pigs. They considered them as their pets – Female full-time pig farmer (NE019, NEFGD3)*.

Animal health workers, when asked to comment on the impact of ASF upon value chain actors all spoke of the emotional toll of depopulation on farmers. While job security and satisfaction were noted by many animal health workers, there were also situations where these workers were vilified as “pig killers” by their communities and felt their personal safety was threatened.

*At the height of depopulation, she would sometimes search her name on Facebook and would find many public posts where she was being labelled as paragadan orig or “pig-killer… The height of the security risk was during the time of depopulation: I was invited inside the farmer's home to sit and talk. I then noticed an itak (bolo knife) below the farmer's chair and recognized it as a threat. This happened three times - one time, the farmer was even holding the itak!”*

–* Interview with animal healthcare worker*.

### Physical Capital

Infrastructure is important for protecting livelihoods from the impacts of infectious diseases. Early on in the epidemic in Camarines Sur, a significant challenge for ASF control was the inability of the local laboratory equipment to test samples, so delaying the receipt of test results. This resulted in depopulation being stalled because they needed a positive result first. This has since been overcome with the laboratory now having the resources for testing.

Beyond laboratory equipment, pigs were an important form of physical capital. While compensation classically involves cash payments, some farmers in this study explained that if their pigs were culled by the government they would prefer replacement pigs of good genetic value. This is because in locations where entire areas are depopulated, high quality pigs may be scarce and cash may be insufficient to assist farmers in recovering from ASF.

### Natural Capital

There were narrative accounts of where community members feared compensation would be insufficient and therefore hid their pigs from government staff until they succumbed to ASF. Carcasses were then discarded in the rivers and elsewhere, polluting the environment. Conversely, following the outbreak of ASF there were some improvements in the management of waterways. The illegal dumping practices exposed underlying mismanagement of waterways by pig farmers. These farmers were banned from keeping pigs beside waterways and some community members celebrated this.

### Transforming Structures and Processes

Laws, regulations and cultural characteristics mediated the impacts of ASF. These were all touched on by a pig trader interviewed in Camarines Sur:

“*He stated that the situation of ASF limited the movement of pigs in Pamplona and increased the supply within the municipality. This is because all the pigs in Pamplona can only be sold to people within the municipality… He also stated that both buying and selling feel very weak because of the ASF situation, and his business was way stronger before. COVID-19 has also made it more challenging since large gatherings are now prohibited by the government. He reminisced that back then, customers would approach him on every special occasion (weddings and fiestas, for example) and they would order three sows! Now, he said, there is no more market for sows – Interview with pig trader (CS-002)*.

**Figure 7:**
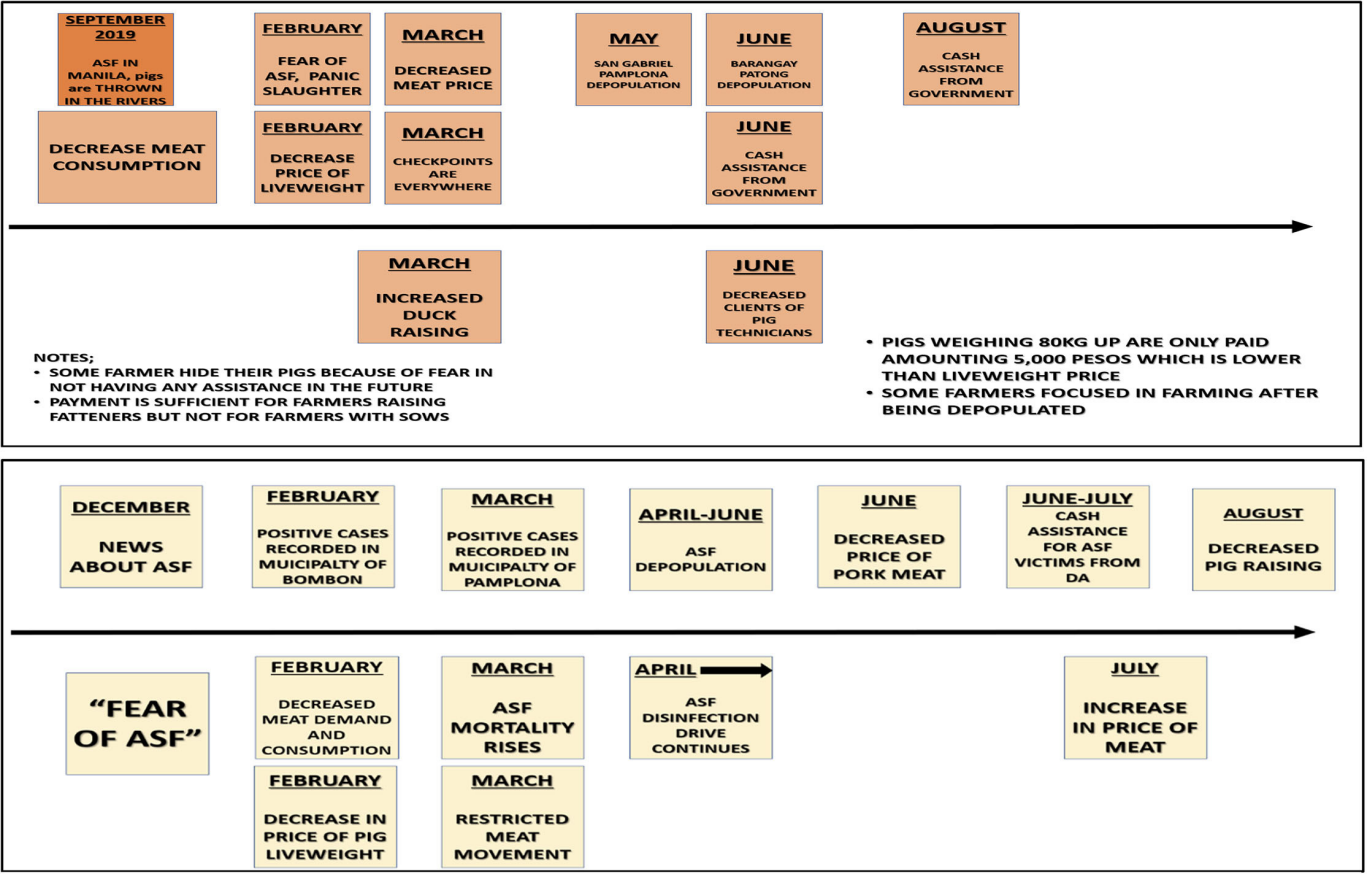
Timelines for African Swine Fever disease events beginning in December 2019, produced during two focus group discussions in Camarines Sur by male (top) and female (bottom) farmers of fewer than 10 pigs.

The depopulation program, including compensation and the program's weaknesses was a topic of passionate discussion. FGD participants included ASF-related information on their community timelines. Participants offered information on deviant behaviour, such as hiding pigs from depopulation teams due to poor communication and resulting fear of insufficient government assistance (Figure 7).

For slaughter, all pigs need a veterinary health certificate (VHC) but according to respondents, regulation was not always successful. In Camarines Sur, the animal health worker believed private veterinarians were issuing VHCs at their clinics without inspecting herds:

*She thinks the poor coordination between private veterinarians and government veterinarians was a challenge. She recalls a story where a pig trader requested Veterinary Health Certificates (six pigs) from a private veterinarian through the phone. This trader told the veterinarian that his pigs came from Milaor (but came from San Fernando where pig mortality was high) and was issued a VHC. This was recognized by the meat inspector (non-veterinarian) in the Naga City Abattoir for slaughter since the raiser presented complete documents signed by a veterinarian. Around 11 pm on that day, one of the pigs died. The remaining five were isolated, and some manifested signs of ASF*.

*So, [the respondent] thinks it is imperative to unite the private veterinarians, government veterinarians, and the consuming public – CS-001*.

This problem was partly addressed by the City Veterinary Office charging much less than private veterinarians (50Php/pig vs. 300Php/pig, respectively) for VHC's which the respondent felt also helped to build relationships between themselves and farmers.

### Livelihood Strategies

**Figure 8:**
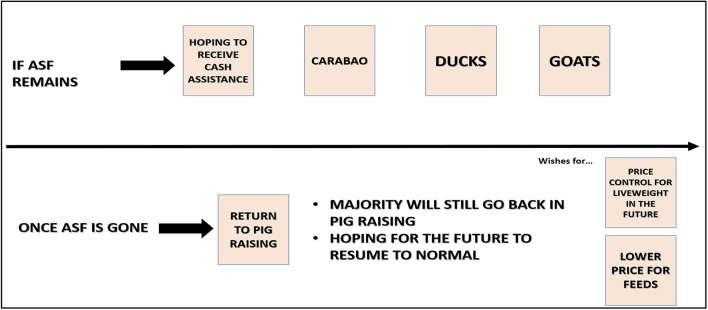
Extension of community timeline into the future to stimulate focus group discussion on planned community responses to African Swine Fever (CSFGD2).

Farmers in FGDs discussed possible community responses to ASF and potential livelihood strategies using a community timeline projected into the future. In Camarines Sur, rather than offering specific dates, participants divided their discussion into two scenarios, one where ASF remains and one where it is eliminated (Figure 8). The discussion was captured by the note-taker, in brief:

*If ASF remains, they hope to receive cash assistance from the government. They will also consider raising carabaos, ducks, and goats instead of pigs. But once ASF is gone, which they pray to happen soon, they would love to return to pig raising. They also wish that liveweight pricing would be uniformed throughout Pamplona and the price of feeds be lowered – Male farmer FGD (CSFGD2)*.

Demonstrations of resilience were captured in the community timelines and open discussions; farmers moved to other livestock species and alternative activities while hoping a vaccine would become available and ASF would be eliminated.

As well as legal practices, animal health workers described deviant behaviour by farmers to counter the personal costs of ASF:

…* some pig raisers would try to secretly slaughter their pigs and even sell to other adjacent barangays. This was possibly influenced by the national campaign stating that ASF-positive pigs are safe to eat since ASF is not zoonotic – CS-001*.

*From the first reported ground zero case at Barangay Santo Niño 1st, there were a total of 166 pigs depopulated. However, there were still some backyard farm owners who transported their herd for slaughter and meat processing despite the policy given by the authority since it was also reported that the ASF virus was not a zoonotic disease. These instances led to the spread of the virus to other barangays in the municipality – NE-001*.

Other value chain actors also adapted livelihood activities. One input supplier started to produce broiler chickens, another began to sell products online and another cut her business expenses. Generally, input suppliers felt optimistic about a return to normal levels of business in the future.

## Discussion

This study describes the broad socioeconomic and livelihood impacts of ASF and the measures used to control it. The study includes findings that can be used to inform future studies and policy measures. All farmers in this study, from small and larger properties experienced financial losses from the ASF epidemic. The financial impacts of ASF on farmers have been described in detail in Africa (19–23); reviewed in (24) and Asia (25, 26). The negative impacts on broader pig value chain players have been described but not quantified in Cameroon (27) and Tanzania (28). Recently the global consequences of a major ASF outbreak in China have been modelled, including knock-on effects on other commodities (29). The impact of ASF on pigmeat markets in Europe has also been recently examined (30). While these numbers provide an important overview of the impact of ASF on agricultural goods, there are fewer published studies on the qualitative impacts of ASF.

In Vietnam, a mixed methods approach was recently used to identify impacts of ASF at value chain, sector and national level and to evaluate the effectiveness of the ASF compensation scheme implemented by the national government (31). Both this study and the Vietnam study mapped impacts along the value chain, but this study placed greater emphasis on qualitative research than the Vietnam study. In addition, this study provides greater detail of the broader livelihoods context of farmers, consistent with the Sustainable Livelihoods Framework (9). This is important because the spread of ASF among smallholder communities in Southeast Asia and the Pacific is superimposed over a complex vulnerability context. Seasonal adverse weather events and corresponding pig disease outbreaks as described in this study are also experienced in other countries inflicted with the disease (26, 32) and these events are only expected to increase with climate change (33). Further, the seasonal peaks and troughs in demand for pork coinciding with ceremonies and cultural events in the Philippines are also seen in other affected countries (32, 34, 35). In these countries, the timing of ASF outbreaks and depopulation programs will influence their impact.

In addition to seasonal vulnerabilities, biosecurity remains a major challenge for smallholder farming systems globally, rendering them more vulnerable to infectious diseases than more biosecure, large-scale production systems. The scale of this disparity has been evidenced with the spread of ASF through Southeast Asia. In China, the proportional rate of outbreaks in smaller farms has been much greater than in medium or large farms (36). The resultant collapse of the smallholder farming sector, which produces more than 80 percent of China's pork, has created big “winners” from the crisis in the form of large pig production firms (35). The shift towards large-scale pig production has also occurred due to ASF in Vietnam (31). As explained by an input supplier in this study, when the millions of smallholders go out of business, the loss of their collective contribution means enormous losses for many associated value chain actors. As the sector becomes dominated by large-scale production there will be concentration of value chain actors, potentially putting many thousands of small-scale input suppliers, buyers, traders, butchers and meat sellers out of business. As observed in this study and as described in peri-urban pig value chains in Uganda (37), biosecurity interventions themselves may also have significant positive and negative impacts on various value chain actors.

Biosecurity challenges extend beyond the farms themselves. In this study, there were accounts of community members hiding sick pigs to avoid their herd being culled. When these pigs died, farmers disposed of the carcasses in rivers. In the Philippines, there is very real concern for the impact of ASF on a native boar species making proper disposal of carcasses a pressing issue (38). The problem of ASF-contaminated carcasses being dumped and contaminating water and waterways has been described in several other countries (27, 39, 40). One of the great challenges of biosecurity and disease control is education. In this study, animal health workers in both sites emphasised the importance of personal communication with farmers. In Camarines Sur, animal health workers described regular contact with every farming household. Given the 1991 devolution of veterinary services in the Philippines (41) and the resource intensity of such a strategy, this may not be replicated in each local government unit.

While a study with a limited sample size of 72 people across two study sites is an insufficient base from which to make policy recommendations, the study findings correspond to several promising interventions warranting further consideration. These were around changes to the ASF control practices and tailoring any support packages to particular needs. The ASF control effort was enormous with all interviewed animal health workers having been recruited to it. The need to consider improvements to the process on the ground became clear. Depopulation campaigns were a dominant theme in discussions, eliciting intense emotion and strong opinions. Farmers had been traumatised by the sounds and vision of their pigs being shot. Pigs were described as pets and even family members by farmers and animal health workers were aware of this, putting them under strain. Many participants were still bearing emotional scars following the loss of their herds and found it difficult to see a future for their livelihood. Human trauma resulting from animal disease control measures appears to be infrequently considered by authorities and academics. Findings in this study echo those described by Mort et al. (42) documenting the psychosocial impacts of the 2001 FMD disaster in the United Kingdom. In their paper, the authors explained that farms are typically places of livestock management and abattoirs the appropriate places for livestock death; depopulation campaigns transgress these boundaries and bring family farmers, who would normally achieve some spatial distancing and emotional detachment into the direct audio and visual experience of the culling. The paper also describes the emotional turmoil of animal health workers and other people on the frontline in the FMD response. This deep and wide-reaching impact of mass depopulation campaigns deserves further attention globally.

As was described as far back as 1985 (27), ASF has the ability to undermine veterinary-farmer relationship, particularly as there is no vaccine or treatment available. In this study, the safety of animal health workers was at risk where depopulation was not supported by the community. While appropriate security measures for animal health workers are important in case of safety breaches, preventative actions, namely improved community engagement processes should be considered. These might also provide an opportunity for delivering information; While biosecurity and prevention measures were not a focus of this study, studies in Timor-Leste (39) and along the Kenya-Uganda border (43) indicate there may be simple interventions such as farmer education, which could be employed to enhance biosecurity and reduce the spread of ASF in smallholder settings.

Improved communication and trust-building with the affected communities will not only improve safety for workers, but it will also likely increase the effectiveness of control programs. Messages around compensation for culled animals need to be timely. In the studied communities, deviant behaviour occurred early in the epidemic before arrangements for compensation were understood by farmers. Information-gathering to determine the most appropriate, desirable form of compensation may also prove useful; Mort et al. (42) echoes the findings in this study, that the loss of a herd of livestock is experienced by some as the loss of their “life's work,” with animal genetics often passed down along family farmer lines along with the intimate knowledge of the farm. This deep loss may be why farmers specifically requested that authorities compensate them with good genetic stock, rather than money.

In addition to better communicating the process of depopulation and compensation with farmers, changes to the culling process should be considered to achieve gentler, more humane practices. This is a situation where a One Welfare approach, acknowledging the interconnectedness of human and animal welfare (44) could be taken; improving pig welfare will have significant impacts on human wellbeing. The significant emotional attachment of Filipino farmers to their pigs is underreported in the literature and from the authors' reading, has not been part of the discussion on ASF in the Philippines.

African Swine Fever does not occur in a vacuum; the impacts it has on communities are augmented by existing livelihood vulnerabilities. The results of this study demonstrate how communities with underlying vulnerabilities such as seasonal changes in income, livestock diseases and natural disasters can be impacted particularly heavily by ASF. A rapid situation analysis such as the one conducted in this study to capture and contextualise ASF impacts within the broader vulnerability context could be used to tailor support according to need.

In addition, value chain actors were impacted by ASF in varied ways. While the overall impact of ASF on society was negative, the network mapping, KIIs and FGDs highlighted the fact that within the value chain, actors were impacted very differently. These pilot data reveal, most actors suffered significant losses as a result of ASF but some actors (certain pig traders) were actually able to increase profits and suffered little or no negative qualitative or quantitative impacts of ASF.

Large-scale/commercial farms may have more of a financial buffer to absorb the economic shock of ASF for longer than smallholders. In the short-term, governments could consider focusing support on backyard farmers with the aim of getting them back into pig raising (as the majority of farmers in this study wanted)—It is important for the value chain that backyard farmers return to pig farming. As explained by a respondent, the losses experienced by the very large backyard farming sector amount to significant losses to other VC actors.

### Limitations

There was an elevation of community transmission of COVID-19 in one of the study sites, Camarines Sur during the pilot and the Department of Health suggested any face-to-face meetings were postponed. As timelines were tight, the research team was quick to adapt. Telephone and web-based interviews were sufficient, and the interview transcripts were full of rich, detailed information but the research teams noted that they could build greater rapport with those interviewees they were able to meet in person.

For the network mapping exercise, which is usually conducted as an in-person, highly interactive multi-stakeholder group activity, some challenges were faced when moving the exercise online in one site. Firstly, participant recruitment was challenging, with fewer people wishing to participate in the online format. Secondly, recruitment was restricted to people with internet access so there was a bias towards better-resourced participants. Thirdly, even for those with internet access, the connection speeds were often slow, and quality was frequently unreliable creating barriers to full participation. The research team rose to the challenge though, conducting follow up calls to clarify details that were missed and try to maximise participant inclusion.

Owing to time and COVID-19 safety constraints, the value chain actors included in the study were limited and did not include either end of the chain, upstream input producers (such as maize farmers) or downstream pork consumers. To increase the external validity of the findings, this study would provide an excellent foundation for development of empirical survey tools as part of an exploratory, sequential mixed methods design, to capture information from many more participants. Additionally, the classical value chain mapping exercises used in this study are unable to assess *ex-*ante the impacts of alternative control scenarios (45). System dynamics approaches are included as an option within the SELIA Framework (4) for conducting impact assessment and modelling ex-ante policy scenarios at the value chain level. These tools were recently applied in the second ASF SELIA study, in Timor-Leste (Jared 5). The selection of SELIA tools for a given livestock disease impact assessment depend greatly on the type of problem being addressed, the time and resources available.

## Conclusion

The availability and accessibility of relevant secondary data in the Philippines is greater than in most ASEAN countries. However, significant gaps exist in the understanding of impact, particularly in qualitative measures. Using multiple participatory tools (network mapping, focus group discussions and key informant interviews), the lived experiences of farmers and other pig/pork value chain actors were captured in rich descriptions. The strength of the qualitative findings was increased using several techniques for triangulation: multiple methods, multiple subjects, comparison with secondary data and semi-quantitative data. These SELIA data provided insights not captured through the use of secondary data and quantitative survey tools. This early application of the SELIA Framework validated the use of classical participatory tools for conducting a rapid socioeconomic and livelihood impact assessment at the community and value chain level. In addition, the network mapping tool showed promise as a first step in a collaborative change-making process, as stakeholders saw the strength in uniting together to help their sector survive ASF.

## Data Availability Statement

The datasets presented in this article are not readily available because access is subject to review by the data custodians. Requests to access the datasets should be directed to d.smith1@uq.edu.au.

## Ethics Statement

The studies involving human participants were reviewed and approved by the University of Queensland Human Research Ethics Committee (Approval Number 2020001543). The patients/participants provided their written informed consent to participate in this study.

## Author Contributions

DS and TC: conceived of and designed the study and contributed equally to drafting and editing the manuscript. DS, TC, and SS: designed the study tools and trained field researchers. MG, MM, MC, LP, RS, and OS: gathered, translated, and transcribed data. All authors contributed to the initial report on which this paper was based and to the paper editing process.

## Funding

This research was funded by the Australian Centre for International Agricultural Research as a Small Research and Development Activity, *Developing a Regional African Swine Fever Socio-economic and Livelihood Impact Assessment Framework* (LS/2019/187).

## Conflict of Interest

The authors declare that the research was conducted in the absence of any commercial or financial relationships that could be construed as a potential conflict of interest.

## Publisher's Note

All claims expressed in this article are solely those of the authors and do not necessarily represent those of their affiliated organizations, or those of the publisher, the editors and the reviewers. Any product that may be evaluated in this article, or claim that may be made by its manufacturer, is not guaranteed or endorsed by the publisher.

## Footnotes

^1^Domingo (2020) 18th August 2020 Update on Zoning Status of Regions in Relation to The Implementation of The National Zoning and Movement Plan for African Swine Fever (ASF), Memo from Office of the Director, BAI (https://www.bai.gov.ph/index.php/regulatory/item/477-asf-portal).

^2^Available online at: BAI ASF Portal https://www.bai.gov.ph/index.php/regulatory/item/477-asf-portal.
