# Supplementary material for: Beyond Numbers: Determining the Socioeconomic and Livelihood Impacts of African Swine Fever and Its Control in the Philippines
Source: Front Vet Sci. 2022 Feb 10;8:734236. doi: 10.3389/fvets.2021.734236 (PMC8866713; doi:10.3389/fvets.2021.734236)
Supplement: Supplementary file 1 [file Table_1.DOCX]

# Sample Focus Group Discussion Plan

Focus group discussions (FGD) can be undertaken with a group of 6-8 farmers in a number of communities at each site. The FGD should not run longer than 3-4 hours. The focus group discussions could be run by a team of around three facilitators and reporters.

Focus group discussions (male and female) for both backyard farming and for specialised farming.

They would concentrate on eight key information gathering activities, in order to develop a clearer picture of the impact of ASF at a community and household level:

**ASF-SELIA Focus Group Discussion –**

**Facilitator Guide**

**Activity 1 Basic Community Information – community timeline**

Materials: AO paper, A6 paper, markers

Outputs: Community timeline; Pig production cards; notes

1. Tell us about your community

*Probes: livestock, cropping, other labour, important event/ceremonies*

2. Tell us about the history of your community (draw timeline from a significant event to now)

*Probes: major events, changes in livelihood activities, COVID-19*

3. What are the different pig production systems within the community? (write down or draw picture to represent)

*Probes: Are these related to different groups within the community, socio-economic status, gender, ethnicity etc*

4. What are Cultural and traditional uses of pigs within the community?

**Activity 2 Overall Livelihood Activities – proportional piling as group**

Materials: AO paper, A6 paper, markers

Outputs: Proportional Piling data; Livelihood activity cards; Livestock species cards, notes

1. What proportion of income generated in this community is generated on-farm vs. off-farm vs. remittances?

*Write livelihood activity cards for on-farm, each type of off-farm work and one for remittances and pile beans. Measure and return to jug.*

2. Of the on-farm income, how much is generated from livestock vs. other activities?

*Pile beans (two piles, livestock and ‘other’), measure and return to jug; Write notes for other on-farm activities*

3. Of livestock, how much is from pigs?

*Write cards for each livestock species and pile beans, measure and return to jug*

**Activity 3 Ranking of importance of livelihood activities – ranking exercise**

Materials: Cards from Activity 2

Outputs: Activity 2 cards in order of importance, notes

1. Ask the group to rank the livelihood activity cards from Activity 2 and ask why they are ranked this way

**Activity 4 Seasonal stressors and opportunities – seasonal calendar**

Materials: AO paper, markers

Outputs: Seasonal calendar, notes – See example next slide

Tell us about your seasons (use paper to draw out seasonal calendar).

Key prompts: *1. Weather*

*2. Food availability*

*3. Labour demands/labour stress periods (men and women separately)*

*4. Key activities for livestock raising (men and women)*

*5. Seasonal challenges of pig production (may include disease)*

*6. Cultural/traditional use of pigs (when? what time of year?)*

**Activity 5 Characteristics of Pig Production & Sales– discussion**

Materials: Pig Production cards from Activity 1; AO paper, markers

Outputs: Discussion notes

1. What are the key characteristics of the pig production system adopted by the farmers in this group?

2. At a household level what are some challenges in pig production?

3. How do you sell your pigs? (i.e. age, buyer, contract arrangements, location, transportation)

*Probes: Who decides when to sell your pigs?*

**Activity 6 Indicative pig production budget: building a picture of typical costs and revenues for this production system**

Materials: AO paper, markers

Outputs: Budget table, notes

1. Reflect on Activity 5 to identify the most typical production system of farmers in the group *note: this does not have to be exactly the production system adopted by one of the farmers in the group, the idea is to think about a typical, usual type of system.*
2. Agree a time period over which to base calculations of costs and revenues – *Note: normally this would be a year, for ease of calculating, but the group could decide on a different timeframe as long as all costs and revenues are calculated consistently*
3. Based on the time period agreed, reach back to activity 5 to identify the stocks and flows of pigs over that time period – number of sows, number of piglets per litter, number of litters per year, total sales per year
4. Identify fixed costs related to pig production – these are costs that do not change when the amount of production changes – for example, loan repayments, insurance, depreciation of assets *note: in an FGD situation these are often very difficult to get an estimation for. if this is the case then ask about a typical loan amount and term and a typical value of housing for pigs. Then the interest repayments and depreciation can be calculated later.*
5. Identify variable costs related to pig production – these are costs that change when the amount of production changes – for example animal feed, veterinary medicines, veterinary services, family labour (male/female), hired labour (male/female) , fuel, electricity, marketing costs. *Note: family labour is usually accounted for as being “free” – in this instance it is important to get an estimate of how many days of family labour are used for pig production even if there is no cash cost associated with this family labour.*
6. Identify revenues related to pig production – quantities and unit value of sales of outputs related to pig production, quantities of outputs related to pig production that are used by household (for example pigs consumed by the household, manure used by household etc.)
7. Information on costs and revenues should be written into a budget table on A0 paper. An example of a budget table for weaner production is given in Table 18.

**Activity 7 ASF Disease Occurrence and impacts: Building on previous activities**

Materials: Community timeline from Activity 1; beans, jug, markers; Budget from Activity 6

Outputs: Building on outputs from Activities 1 and 6, notes

1. Reach back to community timeline and build on it with ASF timeline (starting with emergence in the community), including any responses by farmers and other VC actors, including the animal health department response, any policy changes

2. Proportional piling: Split beans into

i. Two piles - Proportion of pigs got ASF vs not, measure and return to jug

ii. Three piles - Proportion that died, proportion recovered, proportion still sick. Talk about the impacts of this and take notes.

3. Reach back to production budget (Activity 6) and indicate impacts of ASF on these parameters

4. Discuss those impacts beyond the budget – intangible impacts

5. What role (if any) have social networks such as farmer groups played in helping farmers to respond to ASF?

**Activity 8 Looking to the future: building on community timeline**

Materials: Community timeline from Activity 1 again; AO paper, markers

Outputs: Community timeline including looking to the future, notes

1. Extend community timeline to future and discuss any ASF concerns the community foresees and any future plans they have to cope with/respond to them

2. Potential livelihood strategies in response to ASF

*Probes: same ‘business as usual’; changed ways to look after pigs; not having pigs again (e.g. different animal or shift to off-farm labour…)*

Table 18: Example calculation of costs and revenues for weaner production

|  | Quantity | Unit | Unit Value (PHP/Unit) | Total Value per year | Value per weaner sold |
| --- | --- | --- | --- | --- | --- |
| **A. Costs** |  |  |  |  |  |
| **1. Fixed Costs** |  |  |  |  |  |
| Loan repayments |  |  |  |  |  |
| Insurance |  |  |  |  |  |
| Depreciation of assets |  |  |  |  |  |
| **2. Variable Costs** |  |  |  |  |  |
| Hired Labour (male) |  | person days |  |  |  |
| Hired Labour (female) |  | person days |  |  |  |
| Family Labour (male) |  | person days |  |  |  |
| Family Labour (female) |  | person days |  |  |  |
| Animal Feed |  |  |  |  |  |
| Veterinary Medicine and services |  |  |  |  |  |
| Marketing Costs |  |  |  |  |  |
| Fuel |  | litres |  |  |  |
| Electricity |  | kW/hr |  |  |  |
| other costs |  |  |  |  |  |
| **Total Costs** |  |  |  |  |  |
| **B. Revenues** |  |  |  |  |  |
| Weaners sold |  | head |  |  |  |
| Weaners consumed at home |  | head |  |  |  |
| Manure sold |  | tons |  |  |  |
| Manure used at home |  | tons |  |  |  |
| Other revenues |  |  |  |  |  |
| **Total Gross Revenues** |  |  |  |  |  |
|  |  |  |  |  |  |
| **Net Revenue (Gross Revenue - Costs)** |  |  |  |  |  |

# Sample Key Informant Interviews

**ASF-SELIA Interview Guide: Animal healthcare workers**

The AHW interview can be used to gather both personal and community-level information on the impact of ASF

1. **Introduction of yourself and your role**
2. Can you tell us about yourself and about your role in animal health?

*Probes: training, how long lived in the area, changes to role,*

1. **Fees for services**
2. How are animal health services funded?

*Probes: how the AHW charges for services, if the government pays for some services, do they think that the charges mean that only certain groups of farmers can use services*

1. **ASF timeline**
2. When did ASF emerge in your community and what has been the animal health response? (draw out timeline)

*Probes: How did you first find out? What did you do? What instructions did you receive? How did the disease progress? How did the response change over time?*

1. **Strengths/successes in AH response**
2. What have been the strengths of the animal health response to ASF?
3. What has been achieved?

*Probes: control, education, relationship-building, systems strengthening…*

1. **Weaknesses/challenges faced in AH response**
2. What have been the challenges of the ASF response?

*Probes: physical, financial, human, political, human behaviour…*

1. **ASF impact on others**
2. In your opinion, how are different types of farmers affected by ASF?

*Probes: small backyard farmers, larger backyard farmers, commercial farmers – incidence of ASF, costs, income, changes to farming, changes to livelihood*

1. **ASF impact on themselves**
2. What has been the impact of ASF on yourself?

*Probes: human, social, physical, financial, natural (?)*

1. **Hopes for the future**
2. With respect to ASF and animal health more broadly, what are your hopes for the future?

*Probes: Three levels - Most likely to be fulfilled, likely but difficult, biggest dream if no obstacles*

**ASF-SELIA Interview Guide: Input Supplier**

**1: Introduction of the participant’s role** (this can be used as an icebreaker and to gather background information)

1. How would you describe your main role in the pig value chain?

*Probe: what are different parts of the input supply operation – transport, storage, sales, provision of advice, credit…, how many staff are involved*

1. For how long have you been in this role?
2. What other (if any) roles do you play in the pig value chain?
3. How important are these pig value chain roles in your overall livelihood?
4. Has this changed over time?

**2. Understanding Purchasing**

1. What types of products are purchased?

*Probe: different types of inputs relevant to pig production – commercial feeds, feed ingredients, veterinary medicines, equipment etc.*

2. Who do you purchase from?

*Probe: directly from company? From traders? how many sellers of each type of product, any changes over time.*

3. What quantities of each product are purchased?

*Probe: can be on an annual basis, or whatever time period the respondent feels comfortable with. Can probe more about seasonality and differences year-on-year.*

1. What is the price of the products purchased?

*Probe: how has price changed over time?, are there differences in price between different types of suppliers?*

1. What is the relationship with these sellers?

*Probe: long-term, short-term? Any formal or informal contracts, any other services provided to sellers (e.g. credit)*

1. What impact has ASF had on your purchasing?

*Probe: impacts in terms of prices and volumes, immediate impacts vs medium-long term impacts, different impacts related to different types of products/sellers, changes in risk and uncertainty*

**3. Understanding Selling**

1. What types of products are sold?

*Probe: does the input supplier just sell the same products as they buy? Or do they do any transformation – such as blending feeds.*

2. Who do you sell to?

*Probe: should distinguish here between different types of customers –different categories of farmers, traders etc, how many buyers of each category, any changes over time.*

3. What quantities of each product are sold?

*Probe: seasonality and differences year-on-year.*

4. What is the price of the products sold?

*Probe: how has price changed over time?, are there differences in price between different types of buyers?*

5. What is the relationship with these buyers?

*Probe: long-term, short-term? Any formal or informal contracts, any other services provided to you by buyers (e.g. credit)*

1. What impact has ASF had on your selling?

*Probe: impacts in terms of prices and volumes, immediate impacts vs medium-long term impacts, different impacts related to different types of products/buyers/value chains, changes in risk and uncertainty*

**4. Understanding Costs and profits**

1. What are the main costs of your business?

*Probe: understand the key types of cost (labour, rent, fuel etc.) and an estimate of overall costs*

2. Are you able to estimate approximately what your annual profits are?

*Probe: an estimate of overall profits of the business. If the supplier can estimate the proportion related to supplying inputs for pig production this is ideal.*

**5. Impact of ASF on your business**

1. When did ASF start to impact on your business?

2. What was the main immediate impact of ASF on your business?

*Probe: decreased demand and volumes? Changed costs as a result of changed business practices resulting from ASF? Decreased prices?*

3. What has been the main medium - long term impact of ASF on your business?

*Probe: decreased demand and volumes? Decreased prices? Increased costs? Deaths of pigs that you had taken ownership of?*

4. Overall how has ASF affected the profitability and viability of your business?

5. What have been your key responses to ASF impacting your business?

*Probe: reacting to increased uncertainty, changes to business practices, alternative livelihood strategies*

1. **Opinion on impact of ASF on other value chain actors**
2. What is your opinion on impact on small backyard farmers, larger backyard farmers, commercial farmers, traders, input suppliers, other slaughterhouses?

*Probes: incidence of ASF, costs, income, changes to production systems, changes to livelihood*

1. **Hopes for the future**

*Probes: Three levels - Most likely to be fulfilled, likely but difficult, biggest dream if no obstacles*

**ASF-SELIA Interview Guide: Small Scale Slaughterhouse**

1. **Introduction of the participant’s role** (this can be used as an icebreaker and to gather background information)
2. How would you describe your main role in the pig value chain?

*Probe: what are different parts of the slaughtering operation – transport, storage, slaughtering, butchering, sales, provision of advice, credit…, how many staff are involved*

1. For how long have you been in this role?
2. What other (if any) roles do you play in the pig value chain?
3. How important are these pig value chain roles in your overall livelihood?
4. Has this changed over time?

**2. Understanding contracted slaughtering (Ask if slaughtering is a service only and slaughterer does not buy or sell. Otherwise, skip question)**

1. What types of pigs are slaughtered on contract?

*Probe: different types of pigs – ages, weights*

1. Who uses your slaughtering service?

*Probe: do you slaughter on behalf of farmers, traders, butchers? How many actors of each type, any changes over time?*

1. What is the relationship with the people that you slaughter on behalf of?

*Probe: Long-term, short-term? Any formal or informal contracts, any other services provided to sellers (e.g. credit)*

1. What quantities of pigs are slaughtered on contract?

*Probe: can be on an annual basis, or whatever time period the respondent feels comfortable with. Can probe more about seasonality and differences year-on-year.*

1. How much do you charge for slaughtering?

*Probe: how has price changed over time?, are there differences in price between different types of people that you are slaughtering on behalf of?*

1. *What is the form of end product delivered back to the people you slaughter on behalf of?*

*Probe: is it whole carcass, half carcass, butchered cuts? Are there differences between different customers?*

1. What impact has ASF had on your business?

*Probe: impacts in terms of volumes, immediate impacts vs medium-long term impacts, different impacts related to different types of products, changes in risk and uncertainty*

**3. Understanding Purchasing (Ask if the pigs are bought from someone else – so the slaughterhouse takes ownership of the pigs. Otherwise, skip question)**

1. What types of products are purchased?

*Probe: different types of pigs – ages, weights*

2. Who do you purchase from?

*Probe: directly from farmers? From traders? How many sellers of each type of product, any changes over time.*

3. What quantities of each product are purchased?

*Probe: can be on an annual basis, or whatever time period the respondent feels comfortable with. Can probe more about seasonality and differences year-on-year.*

4. What is the price of the products purchased?

*Probe: how has price changed over time?, are there differences in price between different types of suppliers?*

1. What is the relationship with these sellers?

*Probe: Long-term, short-term? Any formal or informal contracts, any other services provided to sellers (e.g. credit)*

1. What impact has ASF had on your purchasing?

*Probe: impacts in terms of prices and volumes, immediate impacts vs medium-long term impacts, different impacts related to different types of products/sellers, changes in risk and uncertainty*

**4. Understanding Selling (Ask if the slaughterhouse takes ownership of the pigs and then sells to others. Otherwise, skip question)**

1. What types of products are sold?

*Probe: distinguish between pork and offal, whole/half carcasses and butchered pork.*

2. Who do you sell to?

*Probe: should distinguish here between different types of customers – traders, butchers, wholesalers, retailers, how many buyers of each category, what type of product each type of buyer purchases, any changes over time.*

3. What quantities of each product are sold?

*Probe: seasonality and differences year-on-year.*

4. What is the price of the products sold?

*Probe: how has price changed over time?, are there differences in price between different types of buyers?*

5. What is the relationship with these buyers?

*Probe: Long-term, short-term? Any formal or informal contracts, any other services provided to you by buyers (e.g. credit)*

1. What impact has ASF had on your selling?

*Probe: impacts in terms of prices and volumes, immediate impacts vs medium-long term impacts, different impacts related to different types of products/buyers/value chains, changes in risk and uncertainty*

**5. Understanding Costs and profits**

1. What are the main costs of your business?

*Probe: understand the key types of cost (labour, rent, fuel etc.) and an estimate of overall costs*

2. Are you able to estimate what a total cost per head of pig slaughtered would be?

3. Are you able to estimate approximately what your annual profits are?

*Probe: an estimate of overall profits of the business.*

4. Are you able to estimate what a total profit per head of pig slaughtered would be?

**6. Impact of ASF on your business**

1. When did ASF start to impact on your business?

2. What was the main immediate impact of ASF on your business? *Probe: change in demand and volumes? Changed costs as a result of changed business practices resulting from ASF? Decreased prices? Policy changes?*

3. What has been the main medium - long term impact of ASF on your business? *Probe: changes in demand and volumes? Decreased prices? Increased costs? Deaths of pigs that you had taken ownership of?*

4. Overall how has ASF impacted on the profitability and viability of your business?

5. What have been your key responses to ASF impacting your business?

*Probe: reacting to increased uncertainty, changes to business practices, alternative livelihood strategies*

**7. Opinion on impact of ASF on other value chain actors**

1. What is your opinion on impact on small backyard farmers, larger backyard farmers, commercial farmers, traders, input suppliers, other slaughterhouses?

*Probes: incidence of ASF, costs, income, changes to production systems, changes to livelihood*

**8. Hopes for the future**

*Probes: Three levels - Most likely to be fulfilled, likely but difficult, biggest dream if no obstacles*

**ASF-SELIA Interview Guide: Pig Trader**

**1. Introduction of the participant’s role** (this can be used as an icebreaker and to gather background information)

1. How would you describe your main role in the pig value chain?
2. For how long have you been in this role?
3. What other (if any) roles do you play in the pig value chain?
4. How important are these pig value chain roles in your overall livelihood?
5. Has this changed over time?

**2. Understanding Purchasing**

1. What types of products are purchased?

*Probe: can distinguish between different ages and types of pigs*

2. Who do you purchase from?

*Probe: should distinguish here between different types of farm production systems (backyard, specialised, commercial), how many sellers of each category, any changes over time.*

3. What quantities of each product are purchased?

*Probe: can be on an annual basis, or whatever time period the respondent feels comfortable with. Can probe more about seasonality and differences year-on-year. With these three questions can build a picture of how much of each different type of product is purchased from which types of sellers.*

1. What is the price of the products purchased?

*Probe: obviously this can be quite a sensitive topic, so some different entry points could be used to discuss – how has price changed over time? Are there differences in price between different types of suppliers?*

1. What is the relationship with these sellers?

*Probe: long-term, short-term? Any formal or informal contracts, any other services provided to sellers (e.g. credit)*

1. What impact has ASF had on your purchasing?

*Probe: impacts in terms of prices and volumes, immediate impacts vs medium-long term impacts, different impacts related to different types of products/sellers/value chains, changes in risk and uncertainty*

**3. Understanding Selling**

1. What types of products are sold?

*Probe: can distinguish between different ages and types of pigs. If the selling product form is different from the buying form then probe about what type of transformation/processing is also done by pig trader.*

2. Who do you sell to?

*Probe: should distinguish here between different types of customers – other traders, farmers, slaughterhouses etc., how many buyers of each category, any changes over time.*

3. What quantities of each product are sold?

*Probe: if there is no holding and transformation, this will be the same as for the purchased quantities. If there are some differences then can probe more about seasonality and differences year-on-year. With these three questions can build a picture of how much of each different type of product is sold to which types of buyers.*

4. What is the price of the products sold?

*Probe: obviously this can be quite a sensitive topic, so some different entry points could be used to discuss – how has price changed over time?, are there differences in price between different types of buyers?*

5. What is the relationship with these buyers?

*Probe: long-term, short-term? Any formal or informal contracts, any other services provided to you by buyers (e.g. credit)*

1. What impact has ASF had on your selling?

*Probe: impacts in terms of prices and volumes, immediate impacts vs medium-long term impacts, different impacts related to different types of products/buyers/value chains, changes in risk and uncertainty*

1. **Understanding Costs and profits**

1. What are the main costs of your business?

*Probe: understand the key types of cost (labour, rent, fuel etc.) and an estimate of overall costs*

2. Are you able to estimate what a total cost per head of pig slaughtered would be?

3. Are you able to estimate approximately what your annual profits are?

*Probe: an estimate of overall profits of the business. If the trader can estimate the proportion related to pig trading this is ideal.*

4. Are you able to estimate what a total profit per head of pig slaughtered would be?

**5. Impact of ASF on your business**

1. When did ASF start to impact on your business?

2. What was the main immediate impact of ASF on your business? *Probe: decreased demand and volumes? Changed costs as a result of changed business practices resulting from ASF? Decreased prices? Deaths of pigs that you had taken ownership of?*

3. What has been the main medium - long term impact of ASF on your business? *Probe: decreased demand and volumes? Decreased prices? Increased costs? Deaths of pigs that you had taken ownership of?*

4. Overall how has ASF impacted on the profitability and viability of your business?

5. What have been your key responses to ASF impacting your business?

*Probe: reacting to increased uncertainty, changes to business practices, alternative livelihood strategies*

1. **Opinion on impact of ASF on other value chain actors**
2. What is your opinion on impact on small backyard farmers, larger backyard farmers, commercial farmers, traders, input suppliers, other slaughterhouses?

*Probes: incidence of ASF, costs, income, changes to production systems, changes to livelihood*

1. **Hopes for the future**

*Probes: Three levels - Most likely to be fulfilled, likely but difficult, biggest dream if no obstacles*

# Network Mapping Exercise Outline and plan

## Introduction

A network mapping exercise would be undertaken with a small group of up to 10 stakeholders from within the municipality including value chain actors, farmers, extension workers and local government officers. The exercise would need 2-3 facilitators and will take around 3 hours. Care must be taken to facilitate the group effectively in order to ensure that one group does not dominate, and voices of all participants are taken into account.

The mapping exercise will result in the development of four interrelated outputs based on the pig value chains in the local area. These provide a first picture of the pig value chains and form the basis of estimation of ASF impacts beyond the farmgate and can later be refined to take into account additional information obtained in farmer focus group discussions and in value chain actor key informant interviews.

Ideally a network mapping exercise would be undertaken face-to-face, with all participants in the same location and able to interact with each other to develop the key outputs in a participatory manner. Face-to-face mapping exercises undertaken pre-COVID-19 (2016) and during COVID-19 (2020) are shown in Figure 30.


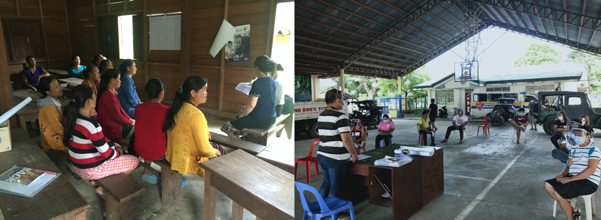


Figure 30: Face to Face Value Chain Mapping/Network Mapping Exercises (l- Cassava Value Chain Mapping in Laos (2016) , r- Pig and pork network mapping in Central Luzon, Philippines (2020))

If it is too risky to undertake face-to-face mapping exercises, then one potential solution is to use online collaboration tools to bring together participants from remote locations (Figure 31). This approach was trialled under the ASF-SELIA Framework Pilot activity in Central Bicol. The team from Central Bicol State University of Agriculture used Google Meet (meet.google.com) as the base videoconferencing tool, Draw Express (drawexpress.com) as an online tool to draw flow maps and used Google Slides (docs.google.com/presentation) to undertake collaborative geographic mapping of the value chain with participants.


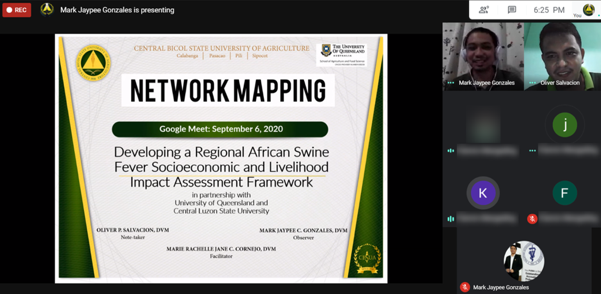


Figure 31. Online Network Mapping Exercise using Google Meet (ASF-SELIA Pilot Activity, Central Bicol, Philippines, 2020)

## Activity 1: Process Matrix

A process matrix is a way of representing the key characteristics of the value chain and value chain actors in an accessible format, as shown in Figure 32.

| Process | Process 1 | Process 2 | Process 3 | Process 4…. |
| --- | --- | --- | --- | --- |
| Actors |  |  |  |  |
| Activities |  |  |  |  |
| Number of Actors and Employees |  |  |  |  |
| Indirect Actors and Service Providers |  |  |  |  |
| Impact of ASF |  |  |  |  |

Figure 32: Example process matrix

The process matrix will be developed in a collaborative manner using facilitated group discussion with the information being recorded on an A0 sheet. Figure 33 shows a participant in a network mapping exercise in Central Luzon, Philippines adding information on activities undertaken by backyard and commercial pig farmers.


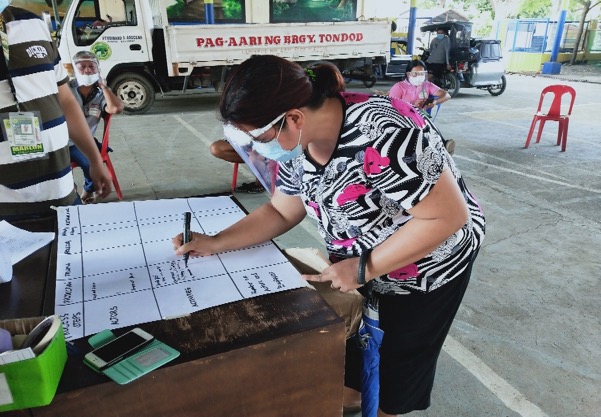


Figure 33: Participant contributing to process matrix development (ASF-SELIA Pilot Activity, Central Luzon, Philippines, 2020)

*Step 1 Identification of Processes* – participants discuss and agree on the core processes that take place in the value chain. Typical processes would include input supply, production, collection, trading, processing, wholesaling and retailing. These processes are recorded in the top row of the process matrix.

*Step 2 identification of Direct Actors* – participants discuss and agree on who are the actors that are directly involved in each process within the value chain. Within each process there can be more than one category of actors – for example “poor farmers, medium farmers, better-off farmers”. The actors are recorded in the second row of the process matrix.

*Step 3 Identification of Activities* – participants discuss and agree on the activities which are actually done by the actors at each process. If there are more than one group of actors defined for a value chain process, then activities should also be defined for each group. The activities are recorded in the third row of the process matrix.

*Step 4 Number of Actors and Employees* – participants discuss and agree on the number of different actors and employees at each of the core processes of the value chain. The number of actors and employees is recorded in the fourth row of the process matrix.

*Step 5 Identify Indirect Actors and Service Providers* – participants identify any *indirect actors and service providers* that link to value chain actors at each process level within the value chain. Where possible, differentiated indirect actors/service providers will be identified for different actor categorizations.

Step 6 *Identify the impact of ASF* – participants describe the impact of ASF of each VC stakeholder group and process level.

Figure 34 shows the final collaboratively developed process matrix developed with value chain actors in Central Luzon, Philippines as part of the ASF-SELIA pilot activities in the Philippines in August 2020.


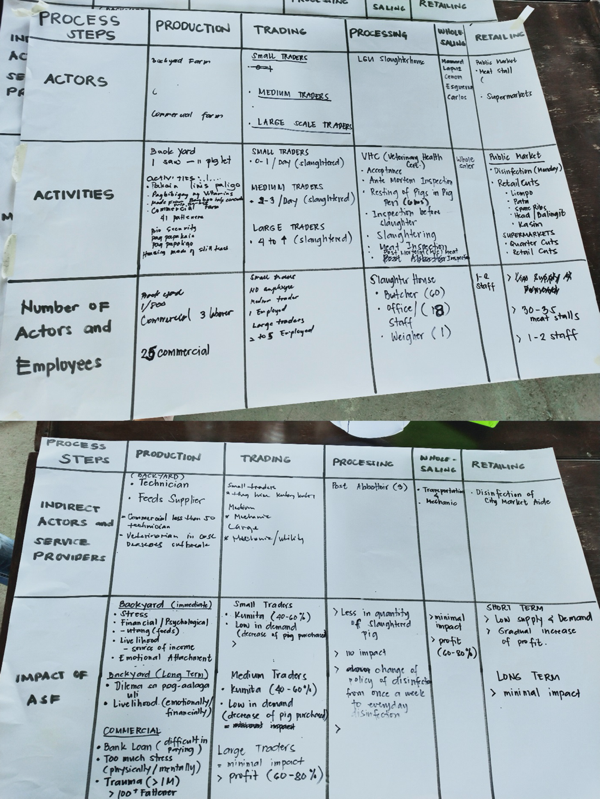


Figure 34: Collaboratively developed process matrix (ASF-SELIA Pilot Activity, Central Luzon, Philippines, 2020)

## Activity 2: Flow, value and relationship mapping

The flow, value and relationship map is developed in a collaborative manner using facilitated group discussion with the following information being included : (i) key categories of actors at each process level and the flow patterns of product between the actors; (ii) the proportional flow of products between actors at each process stage of the value chain; (iii)

the form of each product (for example pigs or pork) to each flow arrow and add unit values of buying and selling at each actor along the value chain; and (iv) information about the relationships between the value chain actors – for example persistent relationships or a spot market relationships added to the map using different line types.


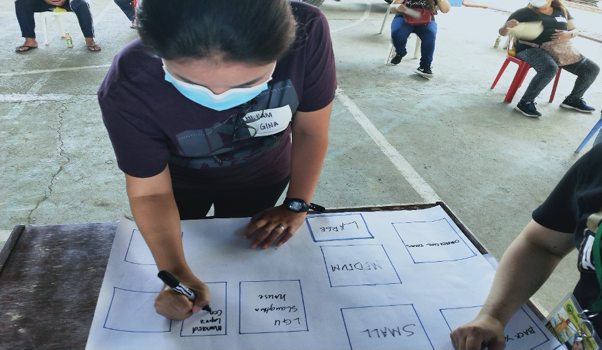


Figure 35: Adding actor names to a flow and value map (ASF-SELIA Pilot Activity, Central Luzon, Philippines, 2020)

*Step 1 Processes and Actors* – Reaching back to the Process Matrix developed in Activity 1, draw processes and different categories of actors on an A0 sheet.
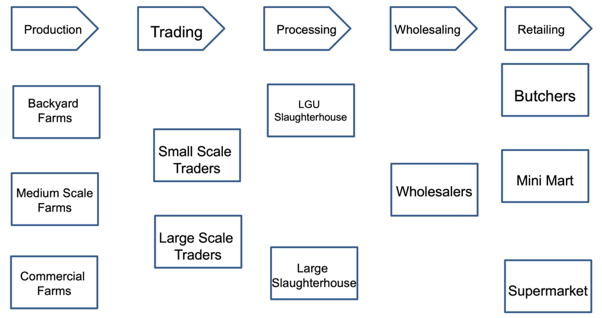


Figure 36: example of including processes and actors in a flow, value and relationship map

*Step 2: Adding volume proportions* – the proportional flow of products between actors at each process stage of the value chain is added to the flow, value and relationship map (see Figure 37). The proportions should add to 100 percent for each process step.


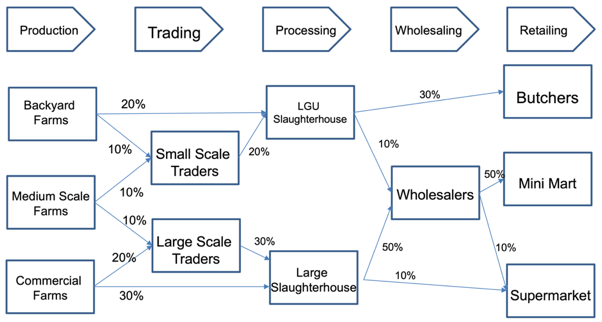


Figure 37: adding proportional flow volumes in a flow, value and relationship map

*Step 3: Adding prices and product forms -* Once the actors and relative volumes of products are mapped, the next stage is to add the form of each product (for example pigs or pork) to each flow arrow and add unit values of buying and selling at each actor along the value chain. This can give an easy indication of the gross margins for each actor. Figure 38 shows a typical example of a flow/value map.


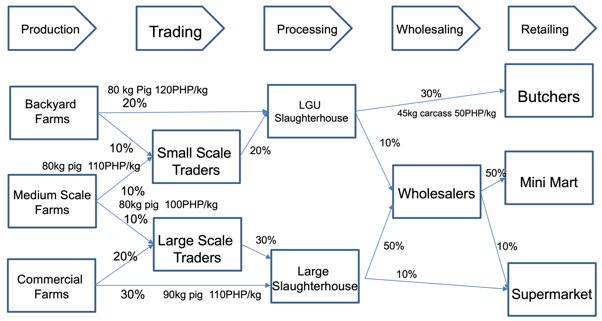


Figure 38: Example flow and value map

*Step 4: adding information about relationships* - The flow/value map can be further enhanced by including information about the relationships between the value chain actors. At the most basic level the relationship could be described as being either a persistent (longer-term, formalized) relationship or a spot market (existing only for a specific transaction) relationship. These differing relationships should be added to the map by the use of different line types (dotted lines for less formalised relationships).

Figure 39 and Figure 40 show the flow and value maps developed during the pilot ASF-SELIA network mapping exercise in Central Luzon, Philippines in 2020. The collaboratively developed map was created on A0 paper and then digitised using the Draw Express program.


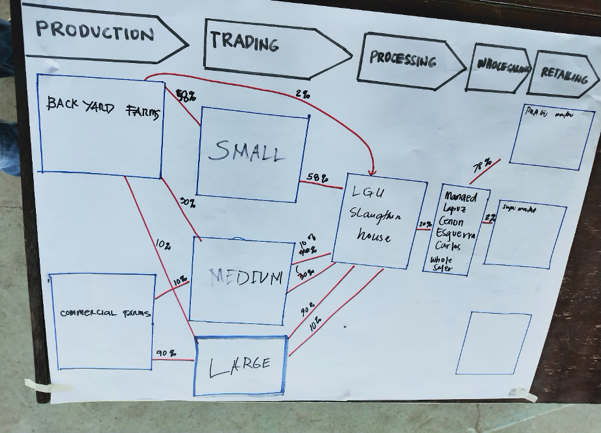


Figure 39: Example relationship map


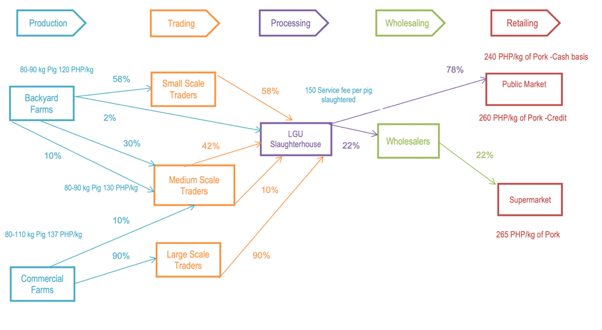


Figure 40: Example relationship map

## Activity 3: Social Inclusion Mapping

Social inclusion mapping of the value chain is undertaken to highlight the heterogeneous nature of actors at various stages of the value chain and to explore the differing characteristics of production, power relations and the differential impact of ASF between social groups.

*Step 1: Identify different groups within a value chain actor category:* Reaching back to step 2 of Activity 1, participants identify different groups within a value chain actor category. For example, rather than using a homogenous grouping (pig farmers), heterogeneous classifications can be developed based on type of production system (backyard or specialised), gender (female and male farmers) or socio-economic status (poor, medium and better-off farmers). These groups are written as the top row of the matrix that makes up the social inclusion map(Figure 41) .

*Step 2: Production Characteristics –* Reaching back to step 3 of Activity 1, participants identify the characteristics of production (for example, the type of housing used for livestock, the type of feed utilised, the overall scale of production) for each group and record in the second row of the matrix.

*Step 3: Power Relations -* Power relations between different social groupings and also between the social group and other value chain actors (for example between poor farmers and input suppliers vs. better-off farmers and input suppliers) are discussed and recorded in the third row of the matrix.

*Step 4: Differential impact of ASF -* Finally, potential impact of ASF on each social group (both positive and negative) is discussed and recorded in the fourth row of the matrix.

|  | Group 1 | Group 2 | Group 3 |
| --- | --- | --- | --- |
| Characteristics of Production |  |  |  |
| Power relations |  |  |  |
| Impact of ASF on each group |  |  |  |

Figure 41: Example Matrix for social inclusion mapping


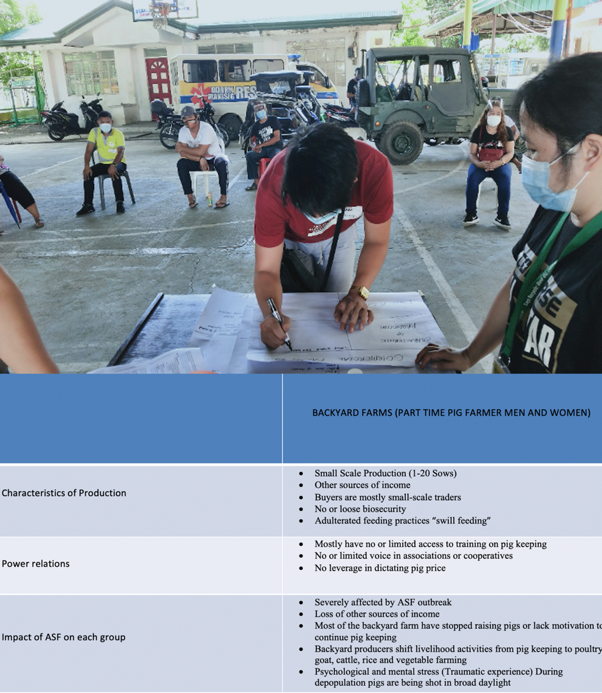


Figure 42: Extract from the Social inclusion matrix developed during the ASF-SELIA Pilot Activity, Central Luzon, Philippines, 2020

## Activity 4: Geographic Mapping

Once the Process matrix, flow, value and relationship map and social inclusion matrix are completed, it is relatively easy to transfer the information to a geographic map of the study region.

*Step 1: Draw Base Map –* with the group, draw a basic geographic map of the study region on an A0 sheet. The map should contain main roads and main administrative boundaries as well as any other key relevant geographic or political features. The map can be drawn freehand, or a map on a computer can be projected onto an A0 sheet taped to a wall and the map traced onto the A0 Sheet (Figure 43).


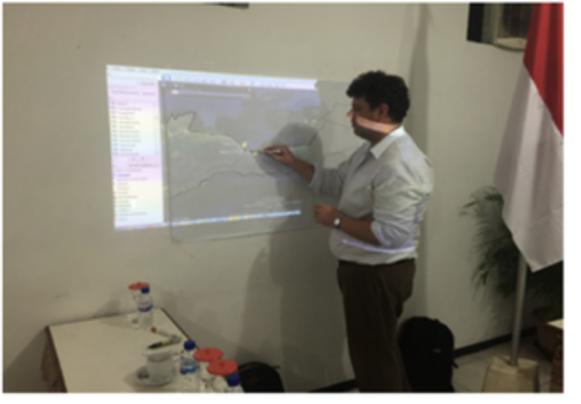


Figure 43: Tracing a map of Bolikhamxay (Laos) onto A0 paper using a projected image.

Step 2: Include key actors and processes - The participants mark the physical locations of the various actors and key processes on the map, as well as an indication of the key product flow routes. Having the key information about the value chain on a geographic map greatly facilitates the organization of any subsequent key informant interviews.


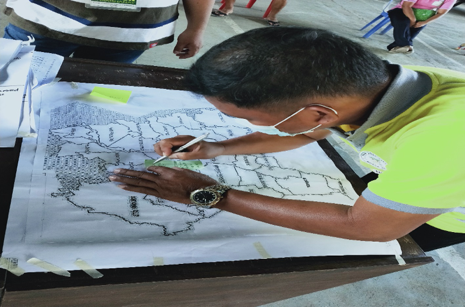


Figure 44: including key actors and processes on a geographic map. (ASF-SELIA Pilot Activity, Central Luzon, Philippines, 2020)

Step 3: Overlay perception of ASF spread – The participants mark where ASF first emerged in the community, how it spread and where it has had greatest impacts on people. The facilitators ask questions about why they think this and write notes. As is the case with the other steps in the geographic map development, this step can be done in person, or can be done using Google Slides as a collaborative tool for participants to overlay information about ASF spread on a pre-prepared geographic map. An example of an overlay generated collaboratively using this technique is shown in Figure 45.


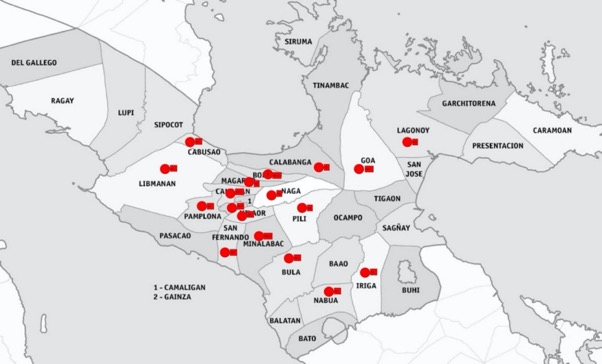


Figure 45: Overlaying perception of ASF spread on geographic map using Google Sheets as an online collaboration tool. (ASF-SELIA Pilot Activity, Central Bicol, Philippines, 2020)

Step 4: Include ASF response – The participants indicate where different actions were taken to control ASF, including actions by individuals (farmers, value chain actors) and actions by government (movement restrictions, testing, culling…etc). An example of this overlay developed collaboratively with the use of post-it notes on a printed map is shown in Figure 46.


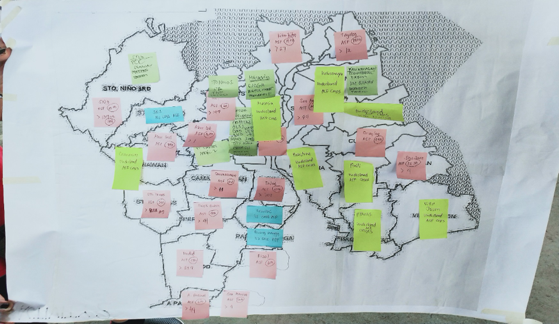


Figure 46: Geographic Map Including ASF response information (ASF-SELIA Pilot Activity, Central Luzon, Philippines, 2020)

The facilitators ask questions about why certain areas targeted and not others and write notes.

Aside from the final 2 steps in activity 4, these activities are based on value chain mapping exercises outlined in Smith et al. (2020). All of the activities have been modified during the pilot testing in Central Luzon and Central Bicol in the Philippines to take into account the realities of implementing during the COVID-19 pandemic. The activities in Central Luzon were undertaken face-to-face, with social distancing measures in place, and the activities in Central Luzon were done entirely online, using collaborative tools.
