# Supplementary material for: Beyond Numbers: Determining the Socioeconomic and Livelihood Impacts of African Swine Fever and Its Control in the Philippines
Source: Front Vet Sci. 2022 Feb 10;8:734236. doi: 10.3389/fvets.2021.734236 (PMC8866713; doi:10.3389/fvets.2021.734236)
Supplement: Supplementary file 2 [file Table_2.DOCX]

## Network Mapping

The **Network Mapping** tool is an enhanced version of value chain mapping that also encompasses elements of sustainable livelihoods and epidemiology. Information gathered in network mapping could include livelihood related information, such as levels of social capital, access to markets and power dynamics. Epidemiological information such as livestock movements and stakeholder biosecurity controls could be collected as well as value chain information such as linkages between actors, prices and volumes and impacts on different actors within the value chain.

Mapping produces a visual representation of the key value chain processes, actors, activities, flows and external influences associated with transforming inputs into agricultural products, through to consumption in end-markets. A value chain map often represents processes for transforming inputs into a commodity or product in a simple linear market segment. In many cases, however, the processes and flows of a commodity or product into multiple markets and subsegments may need to be represented.

Constructing an accurate network map is vital to build understanding the operations of the network system. Accurately mapping these complex systems allows more effective identification of the role of different stakeholders, problems and constraints, and solutions and opportunities.

### Network Mapping in SELIA

Value chain analysis has been utilised as a tool to map network actors and plan control measures beyond the farmgate by gaining an understanding of product flows. This approach was utilised for developing and assessing control measures for Avian Influenza (Antoine-Moussiaux et al., 2017; McLeod et al., 2009; Renard, 2010). More recently, Fournie et al. (2016) and Antoine-Moussiaux et al. (2017) propose using value chain analysis and mapping to more closely examine the behaviour, motives and strategies of value chain actors – and hence to extend the value chain analysis to cover more qualitative and behavioural aspects, in addition to the “traditional” quantitative aspects of value chain analysis.

Within the SELIA framework, Network Mapping will be employed for two main purposes; (i) placing bounds on the scope of the analysis in terms of actors; and (ii) linking the impacts of animal disease on farmers with impacts on upstream and downstream actors.

Mapping the value chain processes and key actors and product flows gives an overview of the key stakeholders within a sector and enables decisions about the **bounds of the analysis** and which actors would most usefully be included within an impact assessment to be made. For example, if an initial value chain mapping exercise identified key actors in the smallholder pig value chain as being (i) feed suppliers; (ii) producers; (iii) traders; (iv) slaughterhouses; and (v) wholesalers, this would facilitate decision making about which actors to include in making an assessment of impacts.

The mapping exercise in SELIA will result in the development of four interrelated outputs based on the value chains in the local area. These provide a first picture of the value chains and form the basis of estimation of disease impacts beyond the farmgate and can later be refined to take into account additional information obtained in farmer focus group discussions and in value chain actor key informant interviews.

| **Example Key Research Questions for ASF**  Some examples of key research questions related to ASF impact assessment that network mapping could contribute to answering include the following:  Which actors are involved in the pig value chain?  What activities do those actors carry out?  How are the pig value chain actors linked to each other?  What are the mechanisms for transmission of impact from farmers to other value chain actors?  What are the direct and indirect impacts of ASF on different actors within the value chain? |
| --- |

A network mapping exercise would be undertaken with a small group of up to 10 stakeholders from within the municipality including value chain actors, farmers, extension workers and local government officers. The exercise would need 2-3 facilitators and will take around 3 hours. Care must be taken to facilitate the group effectively in order to ensure that one group does not dominate, and voices of all participants are taken into account.

The mapping exercise will result in the development of four interrelated outputs based on the pig value chains in the local area. These provide a first picture of the pig value chains and form the basis of estimation of ASF impacts beyond the farmgate and can later be refined to take into account additional information obtained in farmer focus group discussions and in value chain actor key informant interviews.

Ideally a network mapping exercise would be undertaken face-to-face, with all participants in the same location and able to interact with each other to develop the key outputs in a participatory manner. Face-to-face mapping exercises undertaken pre-COVID-19 (2016) and during COVID-19 (2020) are shown in Figure 1.


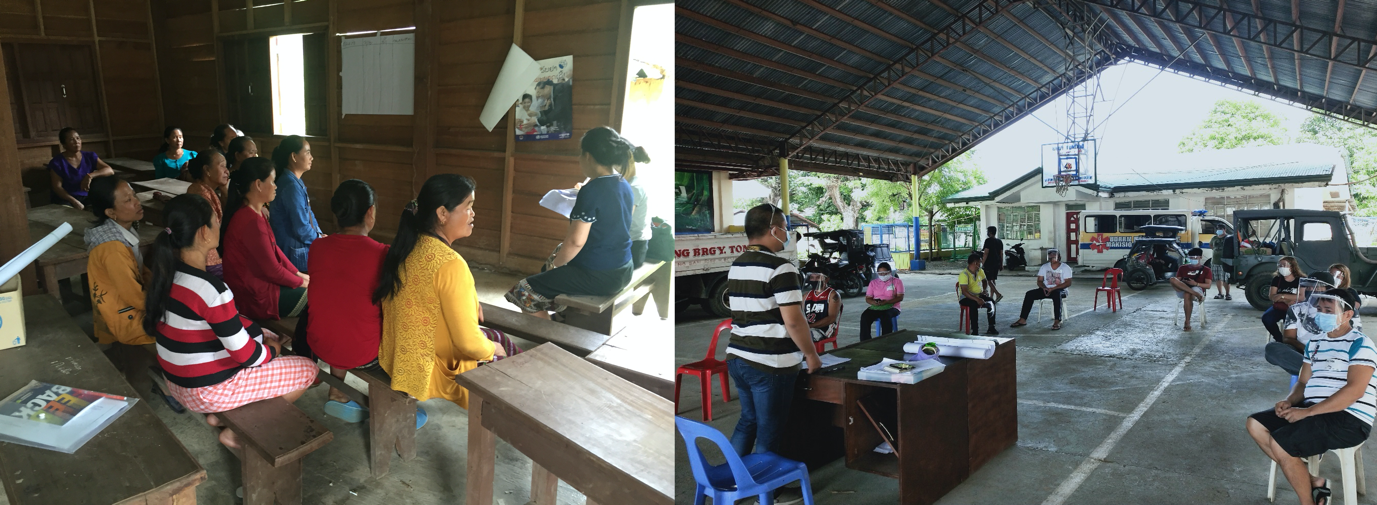


Figure 1: Face to Face Value Chain Mapping/Network Mapping Exercises (l- Cassava Value Chain Mapping in Laos (2016) , r- Pig and pork network mapping in Central Luzon, Philippines (2020))

If it is too risky to undertake face-to-face mapping exercises, then one potential solution is to use online collaboration tools to bring together participants from remote locations (Figure 2). This approach was trialled under the ASF-SELIA Framework Pilot activity in Central Bicol. The team from Central Bicol State University of Agriculture used Google Meet (meet.google.com) as the base videoconferencing tool, Draw Express (drawexpress.com) as an online tool to draw flow maps and used Google Slides (docs.google.com/presentation) to undertake collaborative geographic mapping of the value chain with participants.


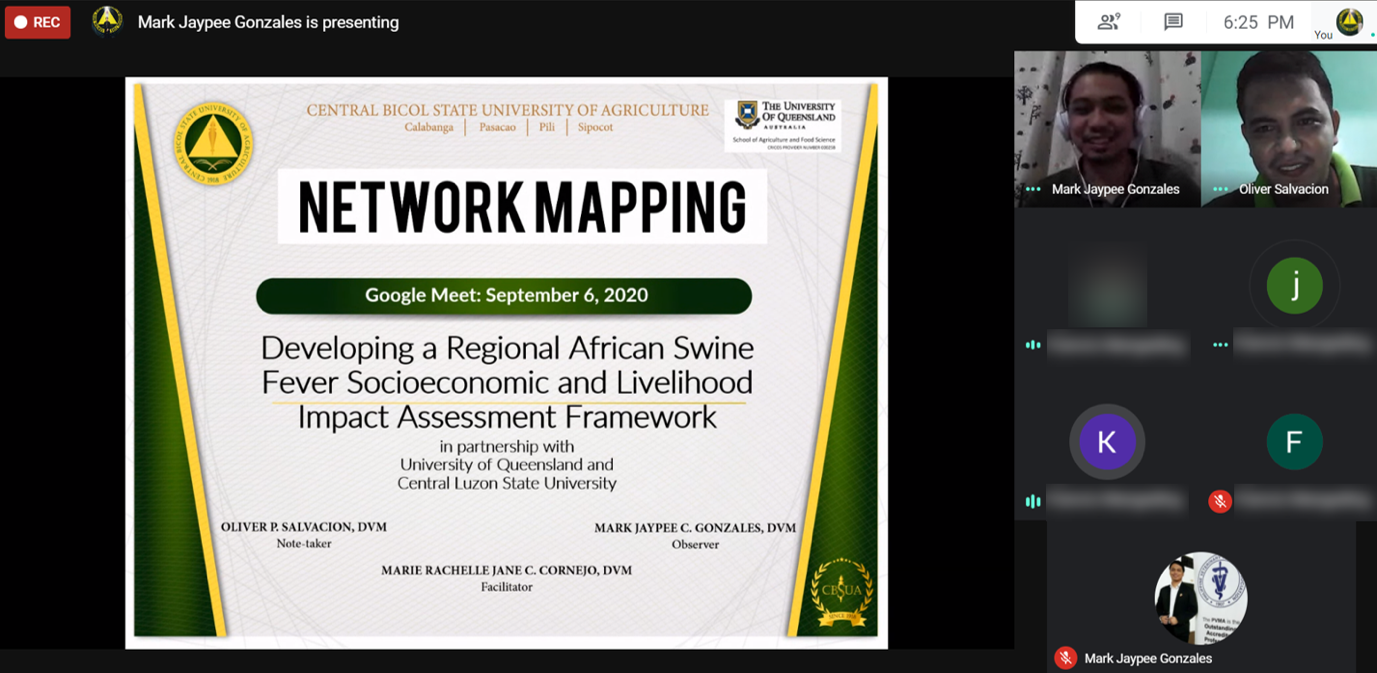


Figure 2. Online Network Mapping Exercise using Google Meet (ASF-SELIA Pilot Activity, Central Bicol, Philippines, 2020)

## Activity 1: Process Matrix

A process matrix is a way of representing the key characteristics of the value chain and value chain actors in an accessible format, as shown in Figure 3.

| Process | Process 1 | Process 2 | Process 3 | Process 4…. |
| --- | --- | --- | --- | --- |
| Actors |  |  |  |  |
| Activities |  |  |  |  |
| Number of Actors and Employees |  |  |  |  |
| Indirect Actors and Service Providers |  |  |  |  |
| Impact of ASF |  |  |  |  |

Figure 3: Example process matrix

The process matrix will be developed in a collaborative manner using facilitated group discussion with the information being recorded on an A0 sheet. Figure 4 shows a participant in a network mapping exercise in Central Luzon, Philippines adding information on activities undertaken by backyard and commercial pig farmers.


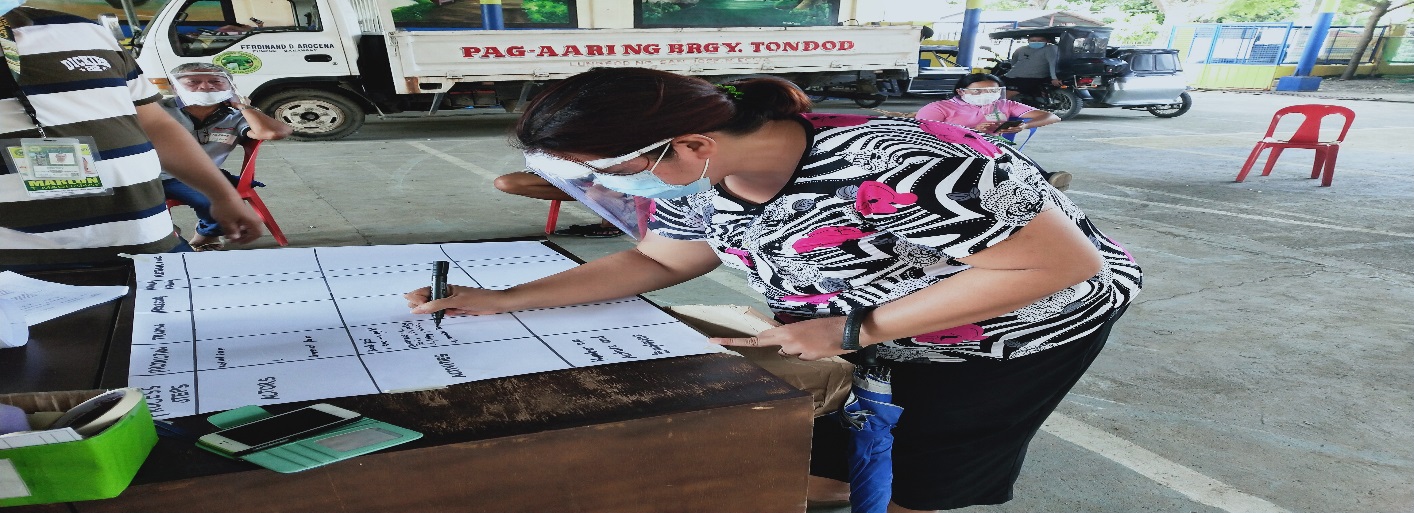


Figure 4: Participant contributing to process matrix development (ASF-SELIA Pilot Activity, Central Luzon, Philippines, 2020)

*Step 1 Identification of Processes* – participants discuss and agree on the core processes that take place in the value chain. Typical processes would include input supply, production, collection, trading, processing, wholesaling and retailing. These processes are recorded in the top row of the process matrix.

*Step 2 identification of Direct Actors* – participants discuss and agree on who are the actors that are directly involved in each process within the value chain. Within each process there can be more than one category of actors – for example “poor farmers, medium farmers, better-off farmers”. The actors are recorded in the second row of the process matrix.

*Step 3 Identification of Activities* – participants discuss and agree on the activities which are actually done by the actors at each process. If there are more than one group of actors defined for a value chain process, then activities should also be defined for each group. The activities are recorded in the third row of the process matrix.

*Step 4 Number of Actors and Employees* – participants discuss and agree on the number of different actors and employees at each of the core processes of the value chain. The number of actors and employees is recorded in the fourth row of the process matrix.

*Step 5 Identify Indirect Actors and Service Providers* – participants identify any *indirect actors and service providers* that link to value chain actors at each process level within the value chain. Where possible, differentiated indirect actors/service providers will be identified for different actor categorizations.

Step 6 *Identify the impact of ASF* – participants describe the impact of ASF of each VC stakeholder group and process level.

Figure 5 shows the final collaboratively developed process matrix developed with value chain actors in Central Luzon, Philippines as part of the ASF-SELIA pilot activities in the Philippines in August 2020.


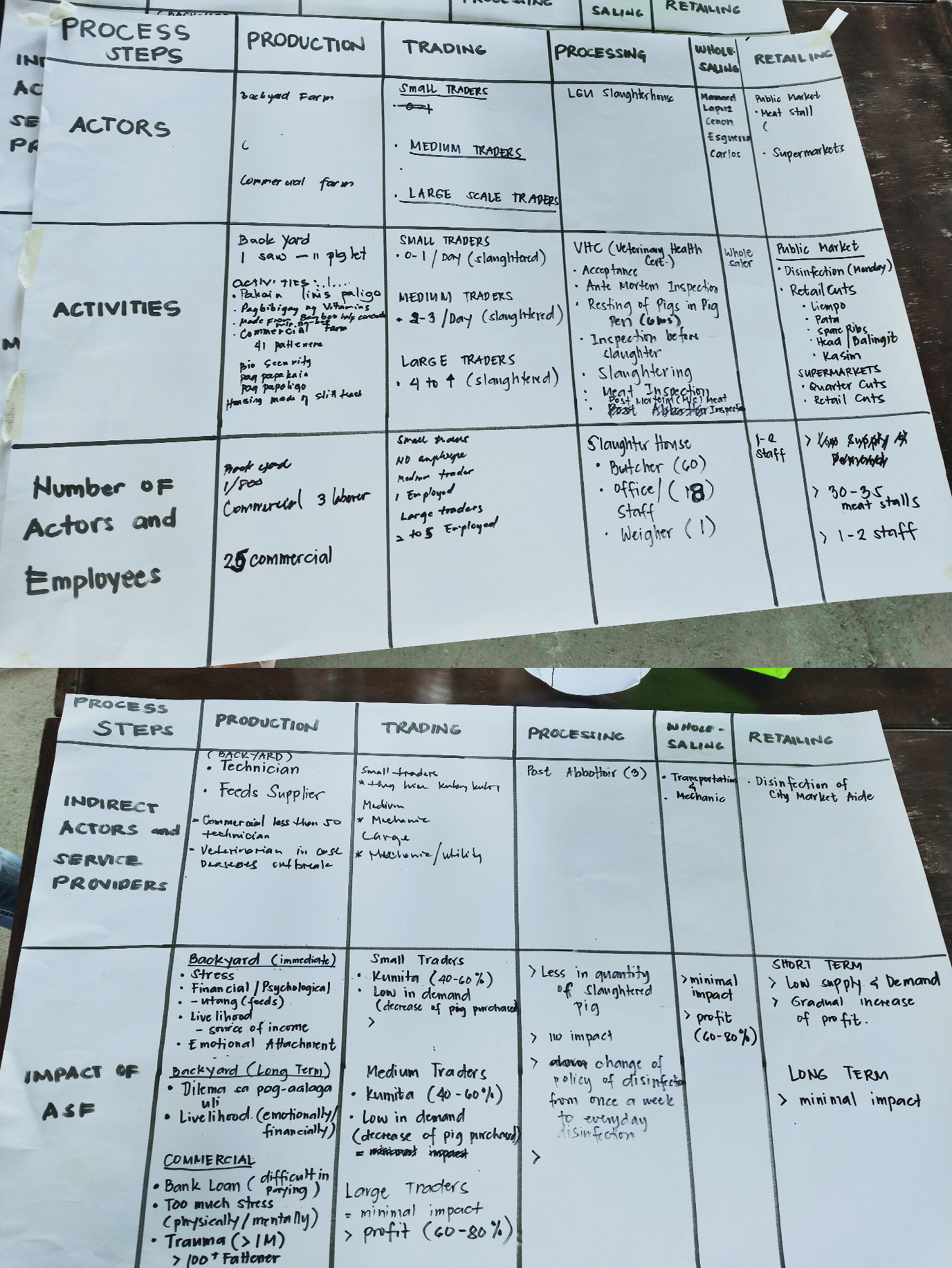


Figure 5: Collaboratively developed process matrix (ASF-SELIA Pilot Activity, Central Luzon, Philippines, 2020)

## Activity 2: Flow, value and relationship mapping

The flow, value and relationship map is developed in a collaborative manner using facilitated group discussion with the following information being included : (i) key categories of actors at each process level and the flow patterns of product between the actors; (ii) the proportional flow of products between actors at each process stage of the value chain; (iii)

the form of each product (for example pigs or pork) to each flow arrow and add unit values of buying and selling at each actor along the value chain; and (iv) information about the relationships between the value chain actors – for example persistent relationships or a spot market relationships added to the map using different line types.


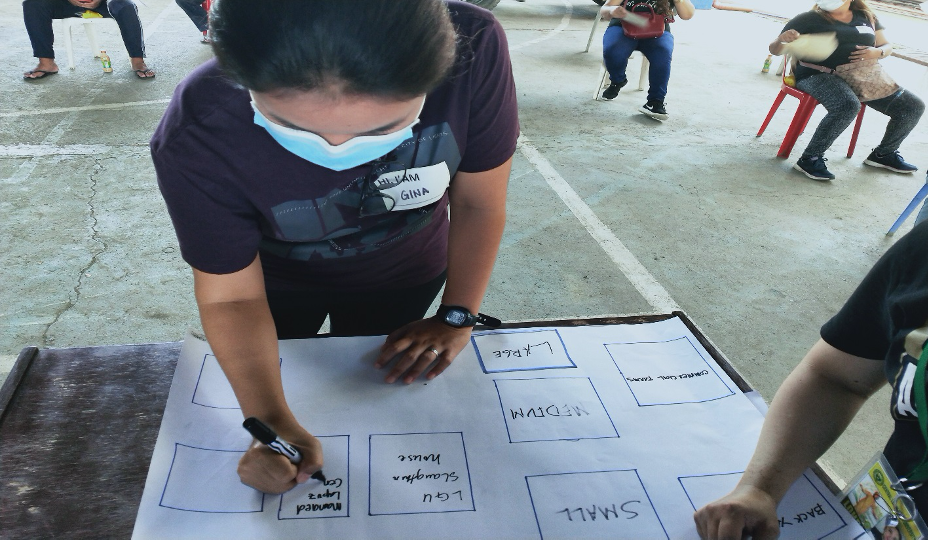


Figure 6: Adding actor names to a flow and value map (ASF-SELIA Pilot Activity, Central Luzon, Philippines, 2020)

*Step 1 Processes and Actors* – Reaching back to the Process Matrix developed in Activity 1, draw processes and different categories of actors on an A0 sheet.
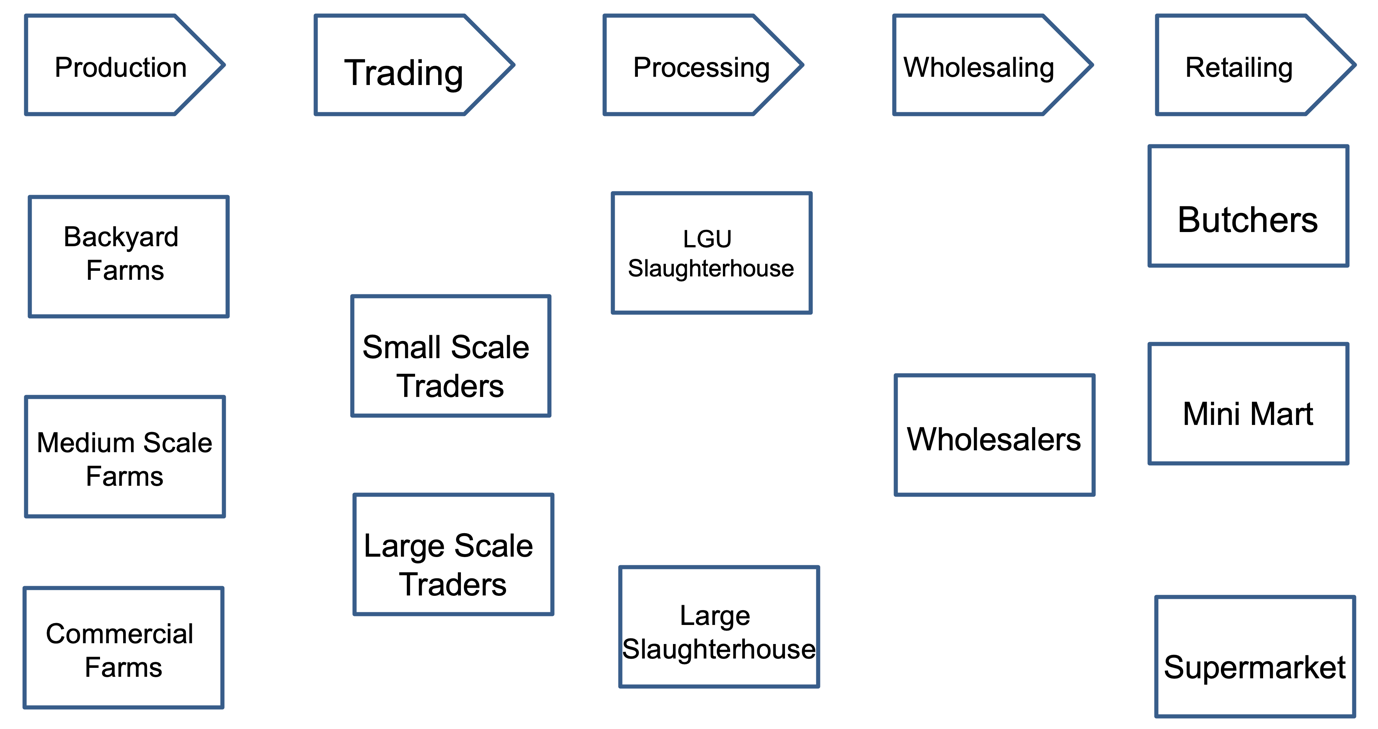


Figure 7: example of including processes and actors in a flow, value and relationship map

*Step 2: Adding volume proportions* – the proportional flow of products between actors at each process stage of the value chain is added to the flow, value and relationship map (see Figure 8). The proportions should add to 100 percent for each process step.


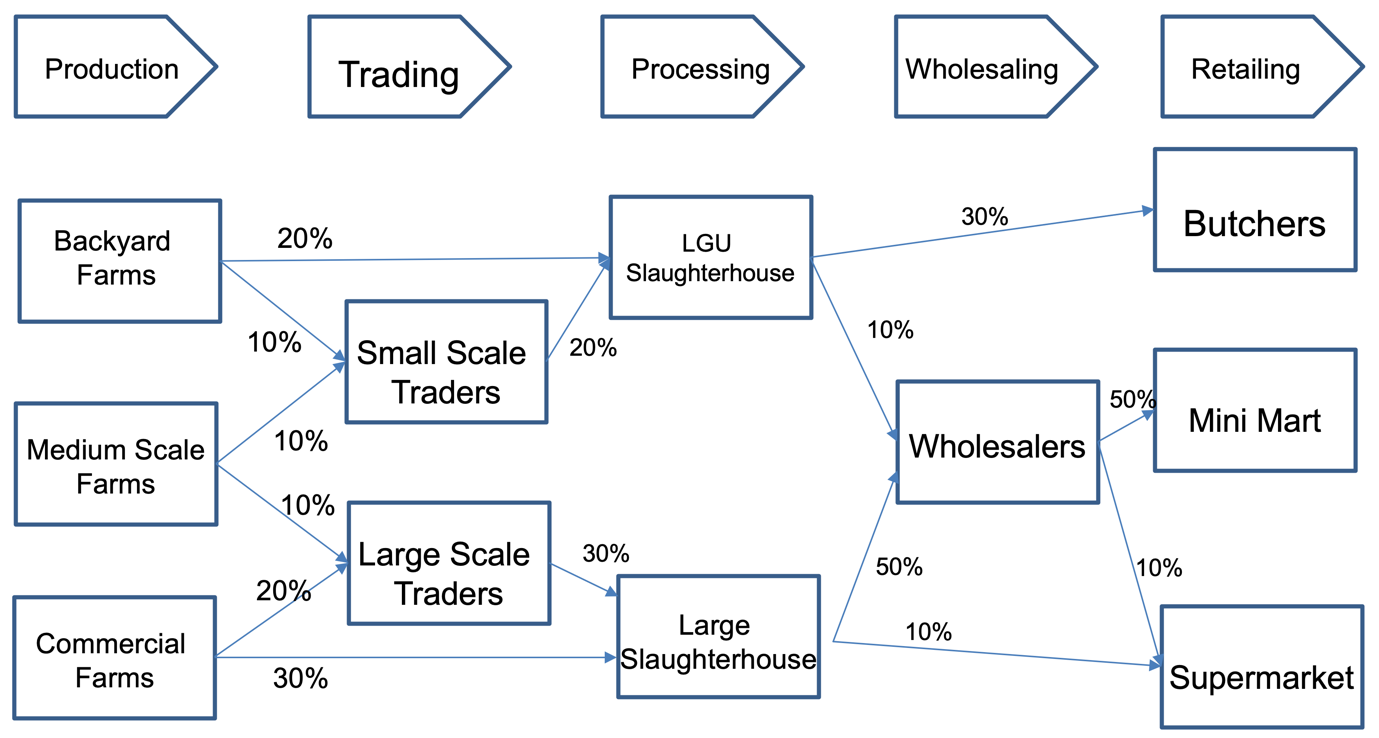


Figure 8: adding proportional flow volumes in a flow, value and relationship map

*Step 3: Adding prices and product forms -* Once the actors and relative volumes of products are mapped, the next stage is to add the form of each product (for example pigs or pork) to each flow arrow and add unit values of buying and selling at each actor along the value chain. This can give an easy indication of the gross margins for each actor. Figure 9 shows a typical example of a flow/value map.


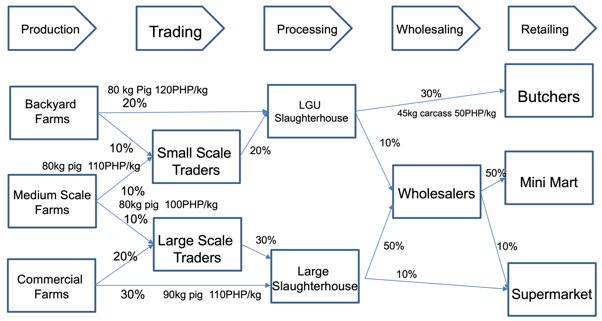


Figure 9: Example flow and value map

*Step 4: adding information about relationships* - The flow/value map can be further enhanced by including information about the relationships between the value chain actors. At the most basic level the relationship could be described as being either a persistent (longer-term, formalized) relationship or a spot market (existing only for a specific transaction) relationship. These differing relationships should be added to the map by the use of different line types (dotted lines for less formalised relationships).

Figure 10 and Figure 11 show the flow and value maps developed during the pilot ASF-SELIA network mapping exercise in Central Luzon, Philippines in 2020. The collaboratively developed map was created on A0 paper and then digitised using the Draw Express program.


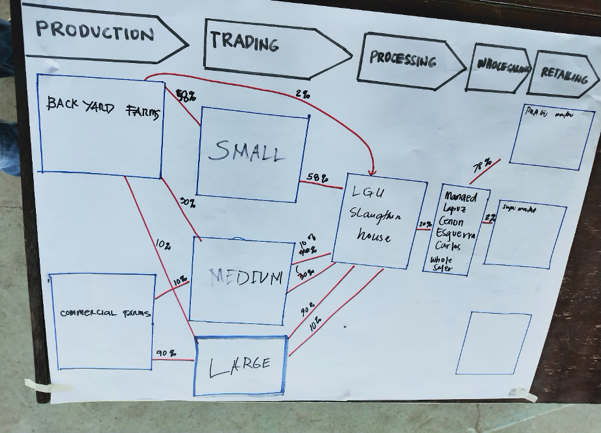


Figure 10: Example relationship map


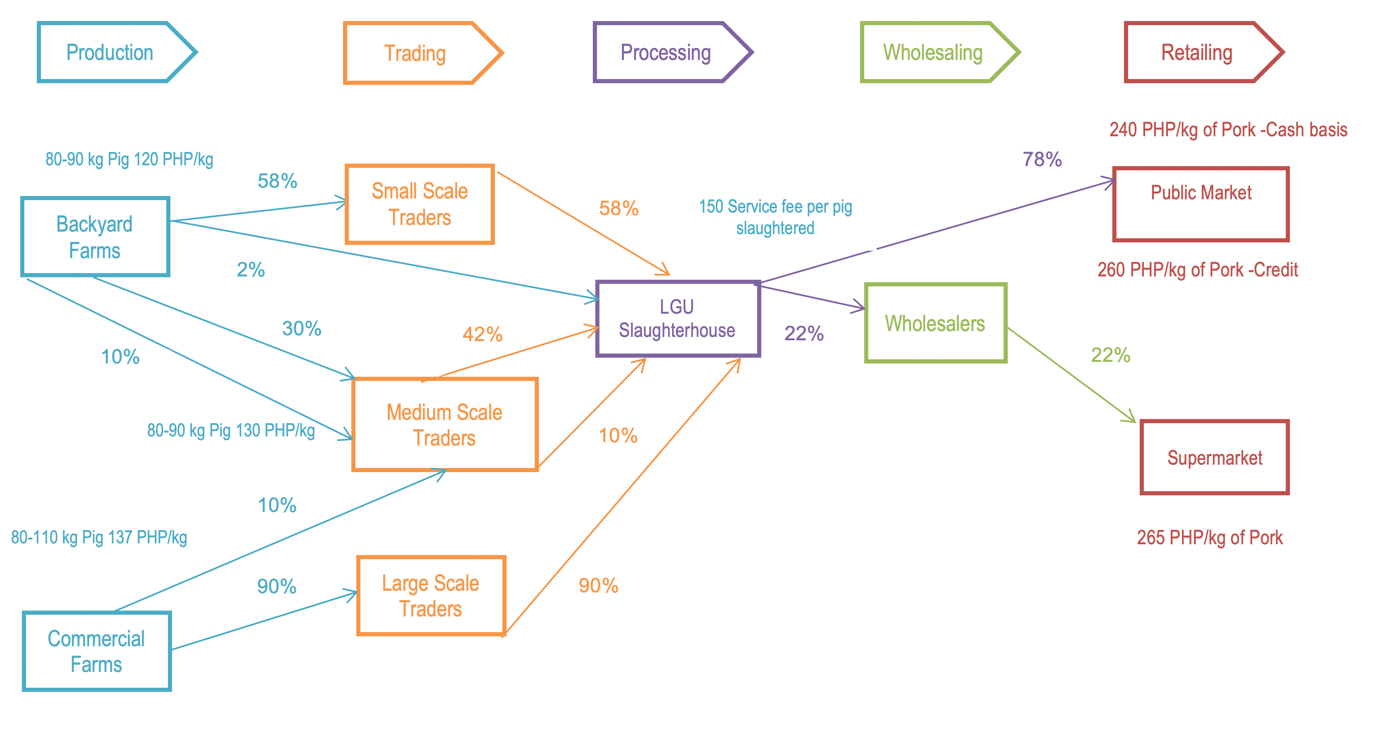


Figure 11: Example relationship map

## Activity 3: Social Inclusion Mapping

Social inclusion mapping of the value chain is undertaken to highlight the heterogeneous nature of actors at various stages of the value chain and to explore the differing characteristics of production, power relations and the differential impact of ASF between social groups.

*Step 1: Identify different groups within a value chain actor category:* Reaching back to step 2 of Activity 1, participants identify different groups within a value chain actor category. For example, rather than using a homogenous grouping (pig farmers), heterogeneous classifications can be developed based on type of production system (backyard or specialised), gender (female and male farmers) or socio-economic status (poor, medium and better-off farmers). These groups are written as the top row of the matrix that makes up the social inclusion map(Figure 12) .

*Step 2: Production Characteristics –* Reaching back to step 3 of Activity 1, participants identify the characteristics of production (for example, the type of housing used for livestock, the type of feed utilised, the overall scale of production) for each group and record in the second row of the matrix.

*Step 3: Power Relations -* Power relations between different social groupings and also between the social group and other value chain actors (for example between poor farmers and input suppliers vs. better-off farmers and input suppliers) are discussed and recorded in the third row of the matrix.

*Step 4: Differential impact of ASF -* Finally, potential impact of ASF on each social group (both positive and negative) is discussed and recorded in the fourth row of the matrix.

|  | Group 1 | Group 2 | Group 3 |
| --- | --- | --- | --- |
| Characteristics of Production |  |  |  |
| Power relations |  |  |  |
| Impact of ASF on each group |  |  |  |

Figure 12: Example Matrix for social inclusion mapping


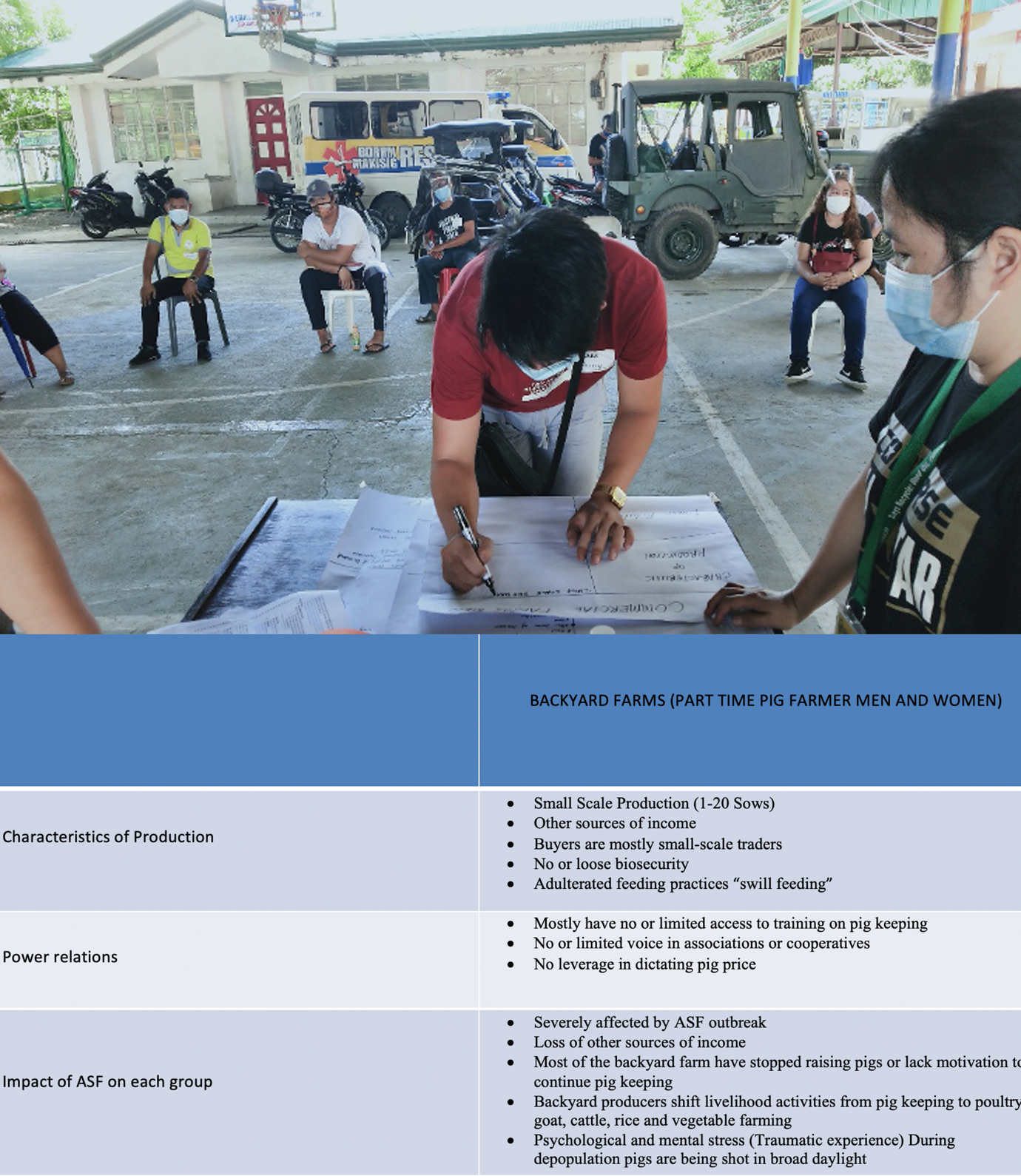


Figure 13: Extract from the Social inclusion matrix developed during the ASF-SELIA Pilot Activity, Central Luzon, Philippines, 2020

## Activity 4: Geographic Mapping

Once the Process matrix, flow, value and relationship map and social inclusion matrix are completed, it is relatively easy to transfer the information to a geographic map of the study region.

*Step 1: Draw Base Map –* with the group, draw a basic geographic map of the study region on an A0 sheet. The map should contain main roads and main administrative boundaries as well as any other key relevant geographic or political features. The map can be drawn freehand, or a map on a computer can be projected onto an A0 sheet taped to a wall and the map traced onto the A0 Sheet (Figure 14).


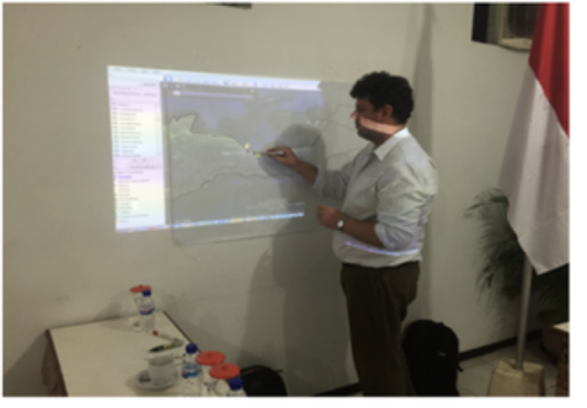


Figure 14: Tracing a map of Bolikhamxay (Laos) onto A0 paper using a projected image.

Step 2: Include key actors and processes - The participants mark the physical locations of the various actors and key processes on the map, as well as an indication of the key product flow routes. Having the key information about the value chain on a geographic map greatly facilitates the organization of any subsequent key informant interviews.


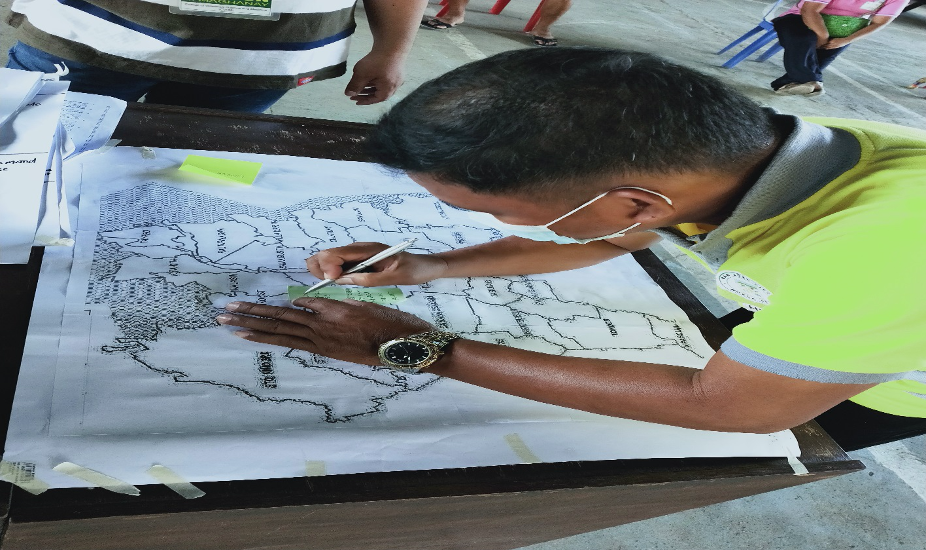


Figure 15: including key actors and processes on a geographic map. (ASF-SELIA Pilot Activity, Central Luzon, Philippines, 2020)

Step 3: Overlay perception of ASF spread – The participants mark where ASF first emerged in the community, how it spread and where it has had greatest impacts on people. The facilitators ask questions about why they think this and write notes. As is the case with the other steps in the geographic map development, this step can be done in person, or can be done using Google Slides as a collaborative tool for participants to overlay information about ASF spread on a pre-prepared geographic map. An example of an overlay generated collaboratively using this technique is shown in Figure 16.


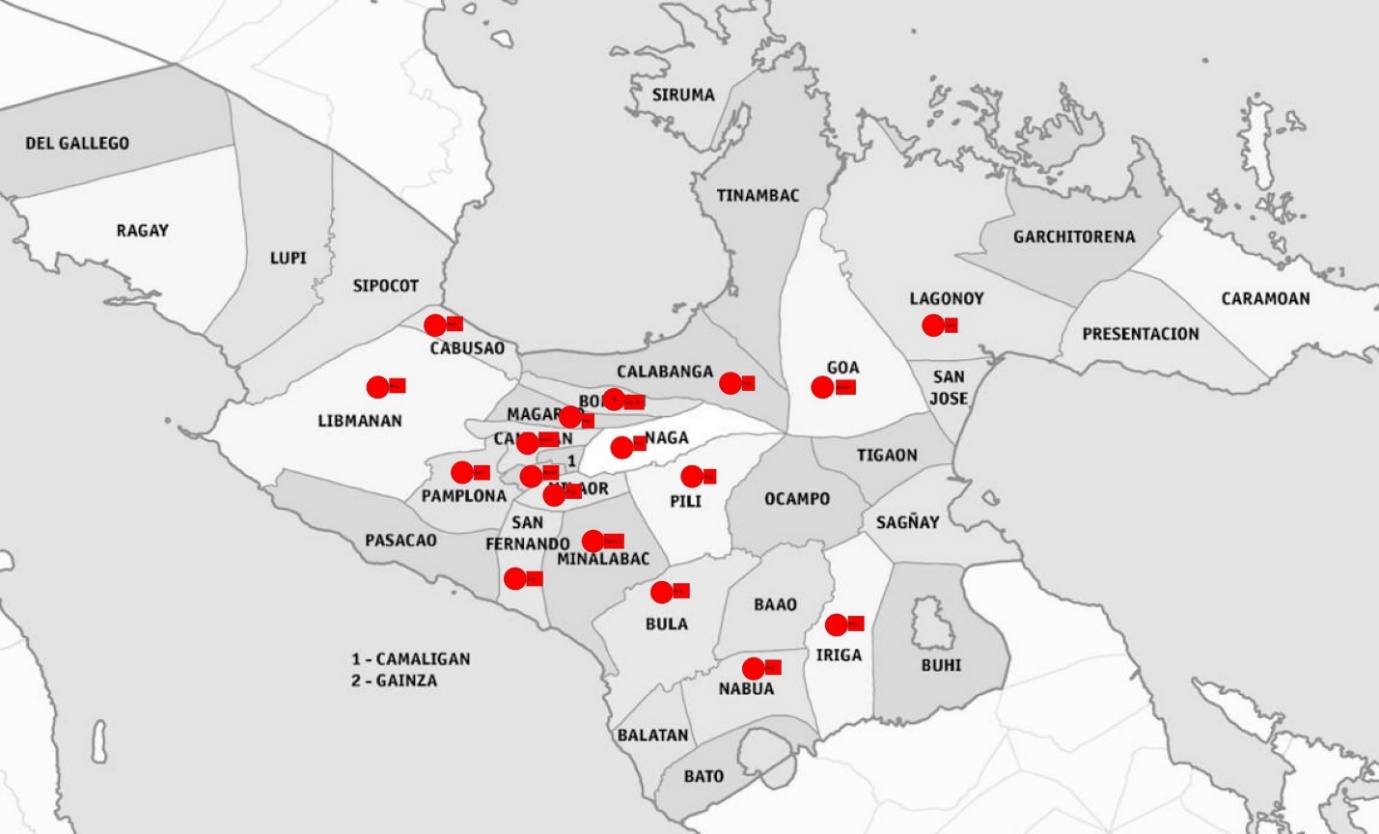


Figure 16: Overlaying perception of ASF spread on geographic map using Google Sheets as an online collaboration tool. (ASF-SELIA Pilot Activity, Central Bicol, Philippines, 2020)

Step 4: Include ASF response – The participants indicate where different actions were taken to control ASF, including actions by individuals (farmers, value chain actors) and actions by government (movement restrictions, testing, culling…etc). An example of this overlay developed collaboratively with the use of post-it notes on a printed map is shown in Figure 17.


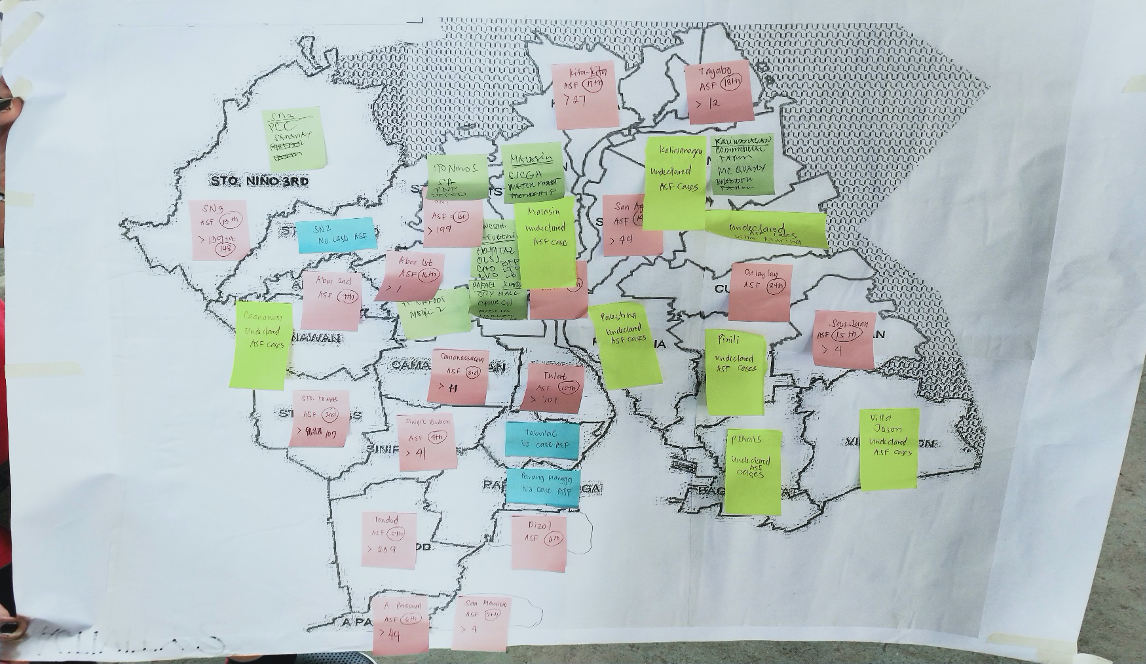


Figure 17: Geographic Map Including ASF response information (ASF-SELIA Pilot Activity, Central Luzon, Philippines, 2020)

The facilitators ask questions about why certain areas targeted and not others and write notes.

Aside from the final 2 steps in activity 4, these activities are based on value chain mapping exercises outlined in Smith et al. (2020). All of the activities have been modified during the pilot testing in Central Luzon and Central Bicol in the Philippines to take into account the realities of implementing during the COVID-19 pandemic. The activities in Central Luzon were undertaken face-to-face, with social distancing measures in place, and the activities in Central Luzon were done entirely online, using collaborative tools.
