# Supplementary material for: Beyond Numbers: Determining the Socioeconomic and Livelihood Impacts of African Swine Fever and Its Control in the Philippines
Source: Front Vet Sci. 2022 Feb 10;8:734236. doi: 10.3389/fvets.2021.734236 (PMC8866713; doi:10.3389/fvets.2021.734236)
Supplement: Supplementary file 3 [file Table_3.DOCX]

# Examples of Analysis of Relevant Secondary Data from the Philippines

A significant amount of data relevant to conducting an impact analysis of ASF is available online from the Philippine Statistics Authority (PSA). We accessed relevant data from the PSA database (<https://psa.gov.ph/pages/survey>) for the two regions where fieldwork was subsequently undertaken - Region III (Central Luzon) and Region V (Bicol Region).

The purpose of secondary data gathering was to support the undertaking of a preliminary quantitative assessment of ASF impacts in the two regions and to identify gaps and ambiguities in the data in order to better guide the fieldwork to gather primary data in the two regions.

1. **Swine Population.**

Quarterly estimates of the overall swine population in both the commercial and backyard sectors are available at the provincial and Regional level. These estimates are developed by the Philippine Statistical Authority (PSA), based on a sample survey of backyard farms and a sample survey of commercial farms.

This enables comparisons of population at backyard and commercial level over time, by region (as shown in Figure 1 and Figure 2) or by provinces within a region. This gives a useful quick overview of the overall sector. However, these comparisons alone do not give insights into the reasons for the changes in population of swine in the different production systems, nor does it give any insight into whether population changes are due to changes in weaner numbers, breeding pig numbers or piglet numbers.


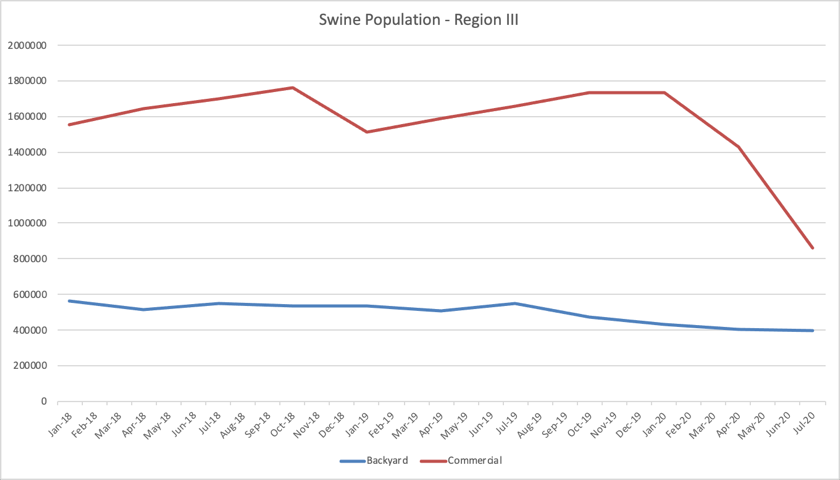


Figure 1: Estimates of Swine Population by backyard and commercial sectors in Region III (Jan 2018 – June 2020)

Source: Backyard Livestock and Poultry Survey (BLPS) and Commercial Livestock and Poultry Survey (BLPS) of Philippine Statistics Authority (PSA).


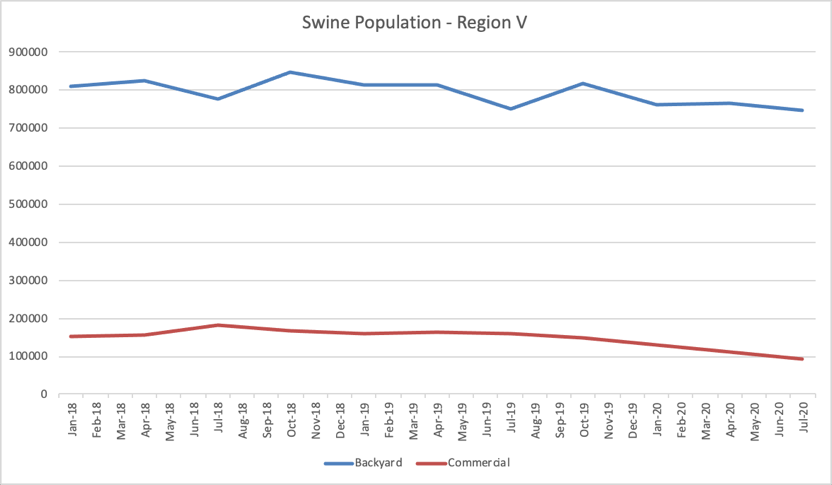


Figure 2: Estimates of Swine Population by backyard and commercial sectors in Region V (Jan 2018 – June 2020)

Source: Backyard Livestock and Poultry Survey (BLPS) and Commercial Livestock and Poultry Survey (BLPS) of Philippine Statistics Authority (PSA).

When estimates of backyard and commercial swine population are presented at regional level (Table 1 and Table 2) a national picture can be built up of the relative changes in composition between backyard and commercial raising that have occurred during a selected time period.

Figure 3 shows a map generated using data from Table 1 and Table 2. The map shows the regional levels of changes in the proportions of pigs begin raised in backyard systems between June 2019 and June 2020. Again, this presents a good overview of the situation in each region, but does not give any insights into the reasons for positive or negative changes in the proportions. In order to present a clearer picture, the swine population data would need to be combined with additional secondary or primary data.

Table 1: Estimates of Swine Population at backyard level by region (2018-2020)

|  | **2018** | | | | **2019** | | | | **2020** | | |
| --- | --- | --- | --- | --- | --- | --- | --- | --- | --- | --- | --- |
| **Backyard** | **January** | **April** | **July** | **October** | **January** | **April** | **July** | **October** | **January** | **April** | **July** |
| **CORDILLERA ADMINISTRATIVE REGION (CAR)** | 201627 | 210878 | 231414 | 247655 | 225941 | 223630 | 256199 | 274403 | 266841 | 252339 | 278126 |
| **REGION I (ILOCOS REGION)** | 471588 | 479600 | 478484 | 482800 | 493894 | 497461 | 484945 | 468197 | 506130 | 467049 | 466746 |
| **REGION II (CAGAYAN VALLEY)** | 412726 | 419748 | 425812 | 429518 | 414617 | 414206 | 411319 | 406356 | 379115 | 366679 | 340444 |
| **REGION III (CENTRAL LUZON)** | 560685 | 514666 | 547970 | 535069 | 538863 | 508615 | 550242 | 472241 | 432619 | 406165 | 395792 |
| **Region IV-A (CALABARZON)** | 389534 | 379854 | 379540 | 403156 | 393677 | 378934 | 377288 | 395207 | 386424 | 374442 | 365556 |
| **MIMAROPA REGION** | 460861 | 471832 | 435857 | 436218 | 432683 | 428381 | 389661 | 393640 | 394278 | 418747 | 395666 |
| **REGION V (BICOL REGION)** | 808240 | 823882 | 775061 | 846348 | 813789 | 812759 | 748547 | 815901 | 760699 | 763356 | 746253 |
| **REGION VI (WESTERN VISAYAS)** | 1103024 | 1123453 | 1118901 | 1162738 | 1109815 | 1113550 | 1083684 | 1124904 | 1075359 | 1078213 | 1060485 |
| **REGION VII (CENTRAL VISAYAS)** | 820129 | 827777 | 816452 | 827615 | 819366 | 831857 | 818615 | 866273 | 845435 | 867317 | 866131 |
| **REGION VIII (EASTERN VISAYAS)** | 275683 | 304901 | 309710 | 310975 | 269264 | 273241 | 287903 | 287903 | 250954 | 250995 | 262693 |
| **REGION IX (ZAMBOANGA PENINSULA)** | 550898 | 522372 | 509610 | 571014 | 568987 | 539369 | 526995 | 598604 | 591216 | 560289 | 532610 |
| **REGION X (NORTHERN MINDANAO)** | 574491 | 590879 | 590278 | 607133 | 589924 | 598124 | 596707 | 612125 | 604132 | 614918 | 620866 |
| **REGION XI (DAVAO REGION)** | 757297 | 748308 | 763022 | 755693 | 760881 | 733561 | 758559 | 752023 | 758536 | 722035 | 742510 |
| **REGION XII (SOCCSKSARGEN)** | 449094 | 459568 | 468010 | 463561 | 467932 | 448968 | 457387 | 460521 | 453060 | 442707 | 444695 |
| **REGION XIII (CARAGA)** | 212548 | 214453 | 216398 | 218679 | 219675 | 210655 | 216533 | 223191 | 216183 | 201450 | 218488 |
| **AUTONOMOUS REGION IN MUSLIM MINDANAO (ARMM)** | 44515 | 47155 | 49874 | 44949 | 48556 | 46791 | 53587 | 48281 | 50419 | 43081 | 65841 |

Source: Backyard Livestock and Poultry Survey (BLPS) of Philippine Statistics Authority (PSA).

Table 2: Estimates of Swine Population at commercial level by region (2018-2020)

|  | **2018** | | | | **2019** | | | | **2020** | | |
| --- | --- | --- | --- | --- | --- | --- | --- | --- | --- | --- | --- |
| **Commercial** | **January** | **April** | **July** | **October** | **January** | **April** | **July** | **October** | **January** | **April** | **July** |
| **CORDILLERA ADMINISTRATIVE REGION (CAR)** | 3637 | 4064 | 3490 | 3412 | 3059 | 3735 | 3929 | 3771 | 4092 | 4083 | 3665 |
| **REGION I (ILOCOS REGION)** | 127555 | 131586 | 127293 | 122374 | 133922 | 147775 | 144429 | 150483 | 156956 | 133925 | 119598 |
| **REGION II (CAGAYAN VALLEY)** | 38281 | 40275 | 40451 | 41561 | 43020 | 42369 | 40261 | 40251 | 41961 | 42454 | 41328 |
| **REGION III (CENTRAL LUZON)** | 1551747 | 1642437 | 1700249 | 1761803 | 1515507 | 1591877 | 1660599 | 1734568 | 1732234 | 1432244 | 859345 |
| **Region IV-A (CALABARZON)** | 1207022 | 1187560 | 1178729 | 1190389 | 1176511 | 1189801 | 1156629 | 1178840 | 1189287 | 1155560 | 1171044 |
| **MIMAROPA REGION** | 133243 | 162742 | 121302 | 128824 | 129188 | 167057 | 125964 | 130708 | 129238 | 168298 | 127395 |
| **REGION V (BICOL REGION)** | 150777 | 157138 | 181379 | 168619 | 161075 | 162962 | 160714 | 148492 | 130758 | 111414 | 94290 |
| **REGION VI (WESTERN VISAYAS)** | 136358 | 142001 | 140185 | 140322 | 140866 | 141936 | 142258 | 146324 | 143875 | 146623 | 148242 |
| **REGION VII (CENTRAL VISAYAS)** | 239666 | 228709 | 236172 | 241054 | 237727 | 235205 | 250524 | 246684 | 262454 | 244012 | 299190 |
| **REGION VIII (EASTERN VISAYAS)** | 9865 | 12396 | 11636 | 12195 | 13146 | 13555 | 14057 | 14141 | 15366 | 15087 | 15985 |
| **REGION IX (ZAMBOANGA PENINSULA)** | 12284 | 10076 | 15768 | 17094 | 10044 | 13655 | 18760 | 20293 | 17316 | 20939 | 21549 |
| **REGION X (NORTHERN MINDANAO)** | 425215 | 409853 | 396426 | 423228 | 431178 | 416511 | 401163 | 446644 | 455188 | 499056 | 492038 |
| **REGION XI (DAVAO REGION)** | 147561 | 147432 | 157581 | 174899 | 181666 | 177146 | 189460 | 182347 | 182720 | 171328 | 159332 |
| **REGION XII (SOCCSKSARGEN)** | 315759 | 324929 | 338736 | 344743 | 348648 | 353199 | 358332 | 352221 | 347374 | 353574 | 368278 |
| **REGION XIII (CARAGA)** | 12531 | 13399 | 14279 | 14032 | 15827 | 15328 | 14741 | 15107 | 15502 | 13644 | 15003 |
| **AUTONOMOUS REGION IN MUSLIM MINDANAO (ARMM)** |  |  |  |  |  |  |  |  |  |  |  |

Source: Commercial Livestock and Poultry Survey (BLPS) of Philippine Statistics Authority (PSA).


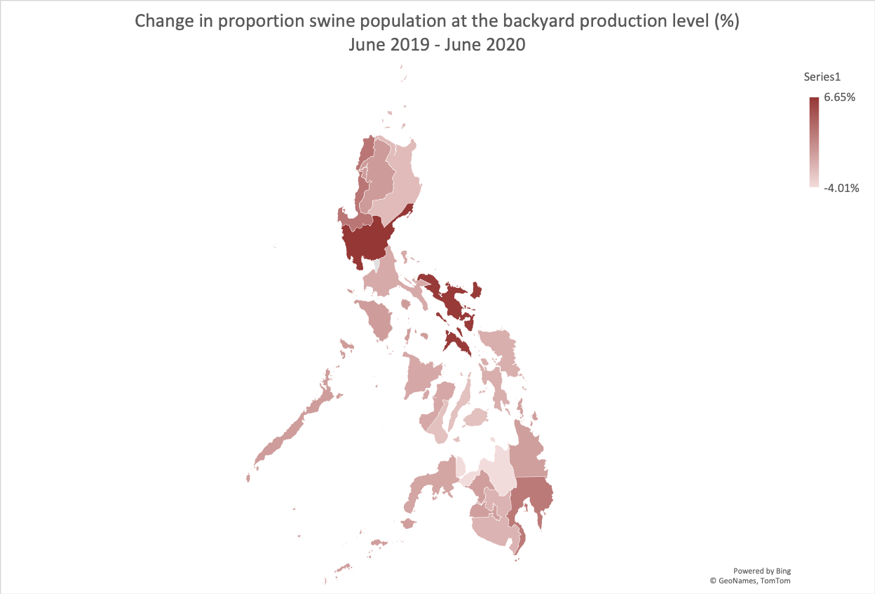


Figure 3: Changes in proportion of swine population raised at the backyard level by region (June 2019 – June 2020)

Source: Generated from data from the Backyard Livestock and Poultry Survey (BLPS) and Commercial Livestock and Poultry Survey (BLPS) of Philippine Statistics Authority (PSA).


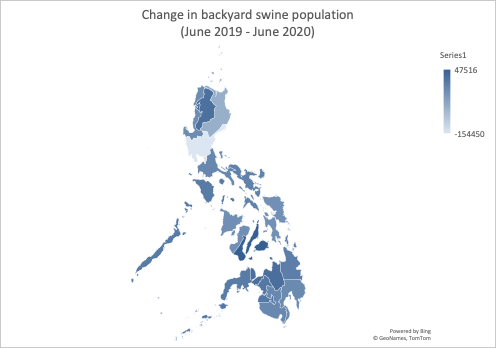


Figure 4: Change in the backyard swine population (head) between June 2019 and June 2020

Source: Generated from data from the Backyard Livestock and Poultry Survey (BLPS) of Philippine Statistics Authority (PSA).

A comparison of Figure 3 and Figure 4 illustrates the need for caution when interpreting swine population data. In Figure 3, Region III is the region with the largest increase in the proportion of swine raised in the backyard – an increase of almost 7 percent over the year. However, as illustrated in Figure 4, in terms of changes in absolute numbers of swine raised at the backyard (and hence potential impact on smallholder farmers), Region III has the largest decline , with almost 155,000 less swine raised in the backyard in June 2020 than in June 2019. More detailed data on the population changes from either the Pork Producers Federation of the Philippines (ProPork) or the National Federation of Hog Farmers, Inc. (NatHog) would be useful to clarify this issue.

1. **Number of hogs slaughtered**

Quarterly estimates of the number of hogs slaughtered in slaughterhouses are available at the provincial and Regional level. These estimates are developed by the Philippine Statistical Authority (PSA), based on surveys of slaughterhouses at provincial level^[[1]](#footnote-1)^ . The data do not distinguish the source of slaughter animals (backyard or commercial), nor do they distinguish the source of the slaughtered animal (within the province/district or from outside). This can hide the true impacts of ASF on different groups of pig producers. Production system disaggregated data were not available from PSA, but may well be available from other sources, including the Philippine Association of Meat Processors, Inc. (PAMPI).


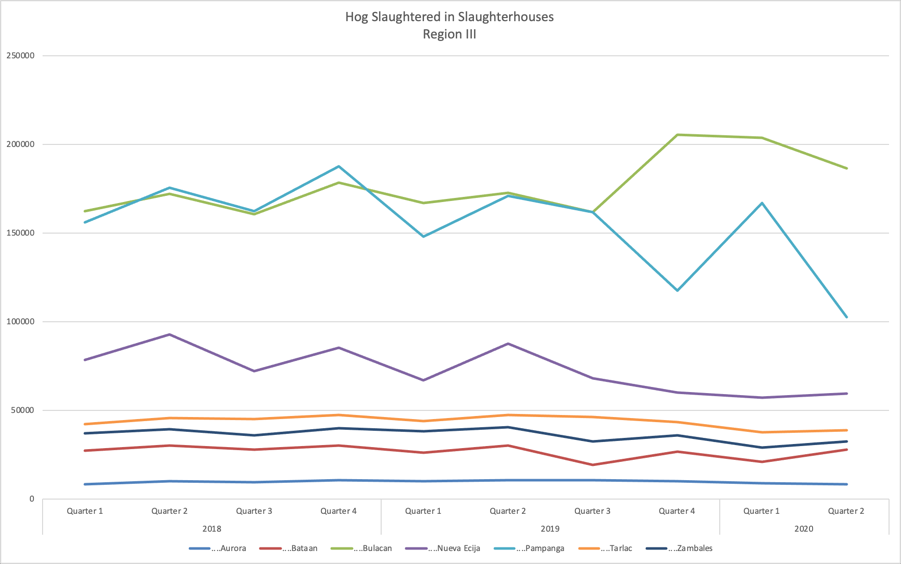


Figure 5: Quarterly estimates of numbers of hogs slaughtered by province in Region III (2018-2020)

Source: Compilation of Data from Survey of Slaughterhouses & Poultry Dressing Plants of Philippine Statistics Authority (PSA).


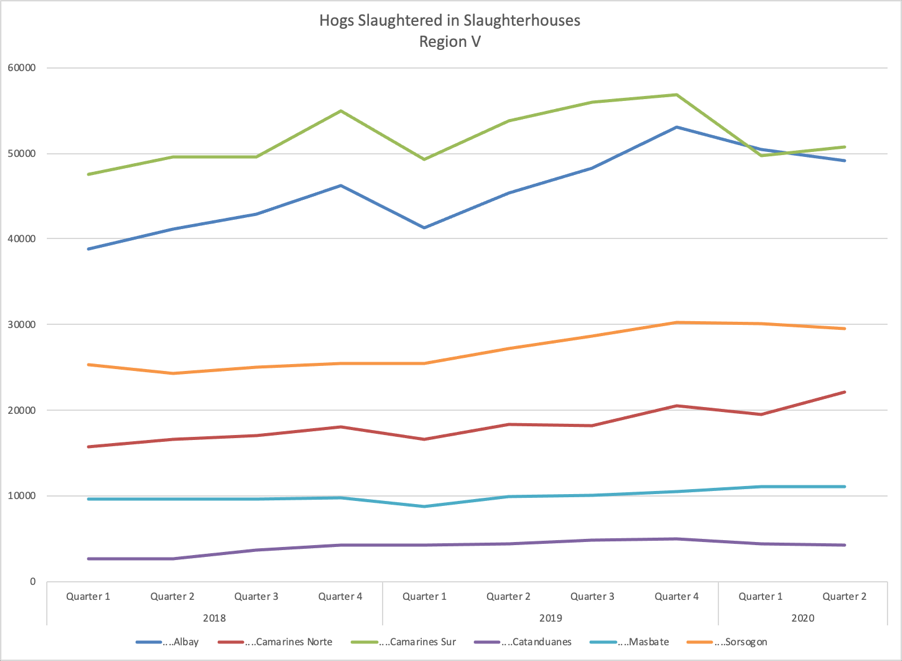


Figure 6: Quarterly estimates of numbers of hogs slaughtered by province in Region V (2018-2020)

Source: Compilation of Data from Survey of Slaughterhouses & Poultry Dressing Plants of Philippine Statistics Authority (PSA).

Table 3: Quarterly estimates of numbers of hogs slaughtered by region (2018-2020)

|  | **2018** |  |  |  | **2019** |  |  |  | **2020** |  |
| --- | --- | --- | --- | --- | --- | --- | --- | --- | --- | --- |
|  | **Quarter 1** | **Quarter 2** | **Quarter 3** | **Quarter 4** | **Quarter 1** | **Quarter 2** | **Quarter 3** | **Quarter 4** | **Quarter 1** | **Quarter 2** |
| **NATIONAL CAPITAL REGION (NCR)** | 317692 | 367924 | 338637 | 364145 | 359943 | 341598 | 325515 | 428668 | 384429 | 319376 |
| **CORDILLERA ADMINISTRATIVE REGION (CAR)** | 50912 | 54501 | 52327 | 56733 | 53600 | 58152 | 55566 | 50319 | 47118 | 44275 |
| **REGION I (ILOCOS REGION)** | 204977 | 225688 | 227092 | 233631 | 217813 | 233584 | 227290 | 213471 | 226294 | 196513 |
| **REGION II (CAGAYAN VALLEY)** | 125786 | 130749 | 132192 | 144972 | 134756 | 142619 | 148701 | 152613 | 144914 | 159250 |
| **REGION III (CENTRAL LUZON)** | 510954 | 565123 | 512970 | 578422 | 499063 | 558515 | 499799 | 498459 | 523007 | 454927 |
| **Region IV-A (CALABARZON)** | 603591 | 661591 | 615909 | 661656 | 573813 | 637121 | 620523 | 584254 | 552615 | 515173 |
| **MIMAROPA REGION** | 75637 | 98699 | 83209 | 87637 | 97903 | 87761 | 87468 | 87660 | 84211 | 85058 |
| **REGION V (BICOL REGION)** | 139480 | 143841 | 147931 | 158566 | 145551 | 158786 | 165901 | 176159 | 165161 | 166888 |
| **REGION VI (WESTERN VISAYAS)** | 175838 | 178978 | 174077 | 186906 | 175283 | 189248 | 192282 | 194924 | 191419 | 185161 |
| **REGION VII (CENTRAL VISAYAS)** | 281341 | 283389 | 294155 | 313389 | 290911 | 304992 | 301816 | 316247 | 283331 | 255785 |
| **REGION VIII (EASTERN VISAYAS)** | 79769 | 86443 | 83491 | 87763 | 79120 | 86258 | 85767 | 88981 | 85281 | 85819 |
| **REGION IX (ZAMBOANGA PENINSULA)** | 45377 | 52527 | 53506 | 73677 | 47121 | 48430 | 58554 | 69104 | 50354 | 44954 |
| **REGION X (NORTHERN MINDANAO)** | 130539 | 139583 | 141564 | 142851 | 124904 | 130179 | 125986 | 142375 | 125343 | 122151 |
| **REGION XI (DAVAO REGION)** | 116395 | 136945 | 140492 | 139257 | 127916 | 128069 | 157164 | 146460 | 126890 | 116440 |
| **REGION XII (SOCCSKSARGEN)** | 101163 | 106612 | 109256 | 119419 | 107380 | 111797 | 121381 | 121645 | 116694 | 112374 |
| **REGION XIII (CARAGA)** | 50850 | 54712 | 52068 | 57069 | 44510 | 52004 | 49055 | 55374 | 40557 | 45342 |
| **AUTONOMOUS REGION IN MUSLIM MINDANAO (ARMM)** | 1937 | 2288 | 2315 | 2936 | 2644 | 2927 | 2964 | 3180 | 2016 | 1765 |

Source: Compilation of Data from Survey of Slaughterhouses & Poultry Dressing Plants of Philippine Statistics Authority (PSA).

1. Volume of Production

Annual estimates of the volume of pig production (turn-off) are available at the provincial and Regional level. The volume of production means the amount of locally-raised animals disposed for slaughter which include animals shipped-out to other regions/provinces. This is expressed in liveweight tons.

These estimates are developed by the Philippine Statistical Authority (PSA), based on surveys of backyard and commercial farms at provincial level. As this information is presented on an annual basis, it is not able to capture the often rapid changes in production that have resulted from ASF – either due to reduced throughput due to pig deaths, or to sudden increases in throughput due to panic slaughtering in advance of ASF. The information on volume of production is also not split up by source of pigs (Commercial or backyard).

However, the data is very useful in order to gain an indication of pre-ASF production levels, and also to combine with information on hog slaughter quantities to gain yield information.

Table 4: Volume of pig production (liveweight tons) by province in Region III (2018-2019)

| **REGION III (CENTRAL LUZON)** | **2018** | **2019** |
| --- | --- | --- |
| **Province** | 472551.76 | 417748.00 |
| **Aurora** | 5271.53 | 6540.89 |
| **Bataan** | 15402.42 | 16218.16 |
| **Bulacan** | 259677.45 | 209776.33 |
| **Nueva Ecija** | 24352.36 | 24109.97 |
| **Pampanga** | 51415.15 | 44189.81 |
| **Tarlac** | 98591.21 | 98628.48 |
| **Zambales** | 17841.65 | 18283.38 |

Source: Backyard Livestock and Poultry Survey (BLPS) and Commercial Livestock and Poultry Survey (BLPS) of Philippine Statistics Authority (PSA).

Table 5: Volume of pig production (liveweight tons) by province in Region V (2018-2019)

| **REGION V (BICOL REGION)** | **2018** | **2019** |
| --- | --- | --- |
| **Province** | 130062.67 | 133246.00 |
| **Albay** | 29400.32 | 31495.01 |
| **Camarines Norte** | 19681.91 | 20497.91 |
| **Camarines Sur** | 43577.48 | 44571.38 |
| **Catanduanes** | 6633.25 | 6645.26 |
| **Masbate** | 15491.37 | 15916.47 |
| **Sorsogon** | 15278.34 | 14121.07 |

Source: Backyard Livestock and Poultry Survey (BLPS) and Commercial Livestock and Poultry Survey (BLPS) of Philippine Statistics Authority (PSA).

1. **Prices of backyard farm production slaughter hogs**

Monthly estimates of the prices of backyard farm production slaughter hogs are available at the provincial and Regional level. These estimates are developed by the Philippine Statistical Authority (PSA), based on a sample survey of backyard farms. Figure 7 and Figure 8 show the monthly backyard farmgate slaughter hog prices in the provinces of Region III and Region V.

Many provinces in both Regions show a “J-shaped” price response curve. The initial onset of ASF led to panic sales and slaughtering and reduced consumer demand for pork – both of which resulted in price declines. After this initial period, the recovery of consumer demand and the tightened supply of slaughter hogs led to price increases. It would be ideal to combine these provincial price time series data with a timeline of ASF outbreaks by province.


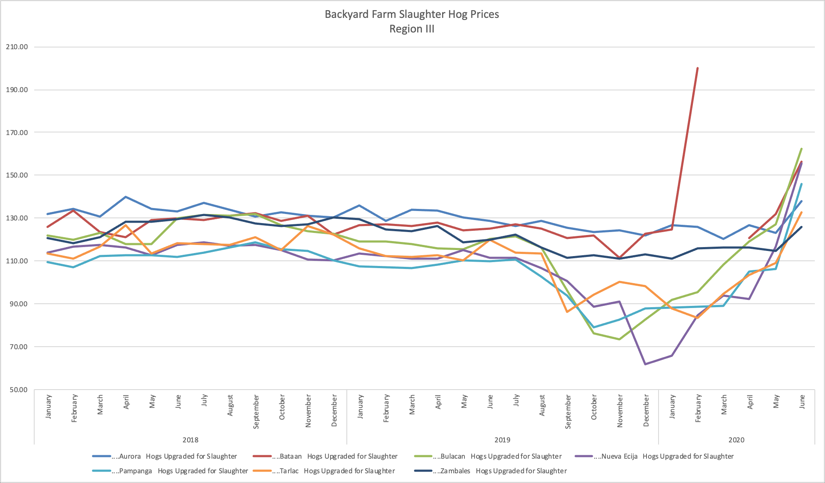


Figure 7: Monthly estimates of slaughter hog prices at backyard level by province in Region III (Jan 2018 – June 2020)

Source: Farm Prices Survey (FPS) of Philippine Statistics Authority (PSA).


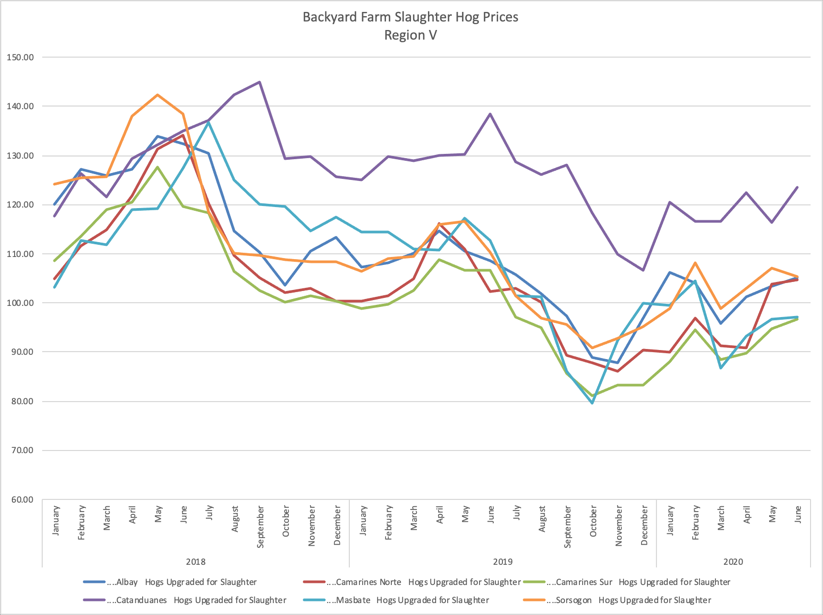


Figure 8: Monthly estimates of slaughter hog prices at backyard level by province in Region V (Jan 2018 – June 2020)

Source: Farm Prices Survey (FPS) of Philippine Statistics Authority (PSA).

1. Preliminary Analyses

By combining the data on the farmgate prices of slaughter hogs and the figures for number of hogs slaughtered, a picture of the monthly value of slaughtered pigs for different provinces can be built up. In order to derive these monthly estimates a number of assumptions need to be made:

1. Farmgate prices are available on a per kg basis. In order to calculate a per head price for slaughter pigs, we assume that the average slaughter weight of pigs in Region III is 80kg (based on information from PCAARRD)
2. Prices are available on a monthly basis, but slaughter numbers are available on a quarterly basis. For the purposes of this exercise we assume that the slaughter numbers in each province are distributed equally amongst the three months of the quarter.
3. The proportion of slaughtered pigs from backyard and commercial levels in each province are not given in the statistics. We assume that the proportions of pigs slaughtered from each sector are the same as the proportions of overall swine population in the province in the same time frame.
4. Differentiated prices for slaughtered pigs at backyard and commercial levels are not given. For this analysis we assume that the farmgate prices are the same for both the commercial sector and the backyard sector.


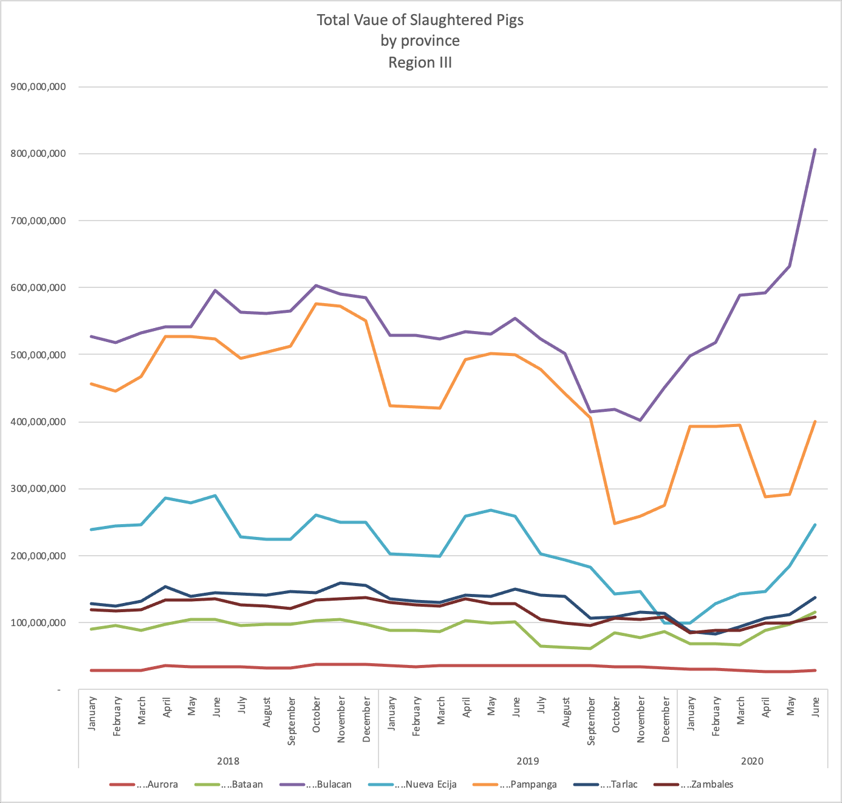


Figure 9: Estimated Total monthly value of pigs slaughtered by province in Region III (Jan 2018 – June 2020)

Source: Farm Prices Survey (FPS) and Survey of Slaughterhouses & Poultry Dressing Plants of Philippine Statistics Authority (PSA).

The initial analysis shows that in many provinces of Region III, the overall monthly value of pigs slaughtered in June 2020 is higher than in most months between January 2018 and May 2020. The exception to this is Pampanga, where the monthly value of slaughter is still significantly lower that in the pre-ASF period. In Bulacan, the slaughter value has increased steadily since the third quarter of 2019 and has doubled during that period.

Separating by production system gives more insight into what is happening in each province. As Figure 10 shows, the value of slaughtered pigs at the backyard production system has remained relatively stable for most provinces, with declines in numbers in 2019-2020 being offset by increases in prices. However, the major exception is Pampanga – where the value of slaughtered pigs from the backyard sector declined significantly during Q3 2019 and has not yet fully recovered.


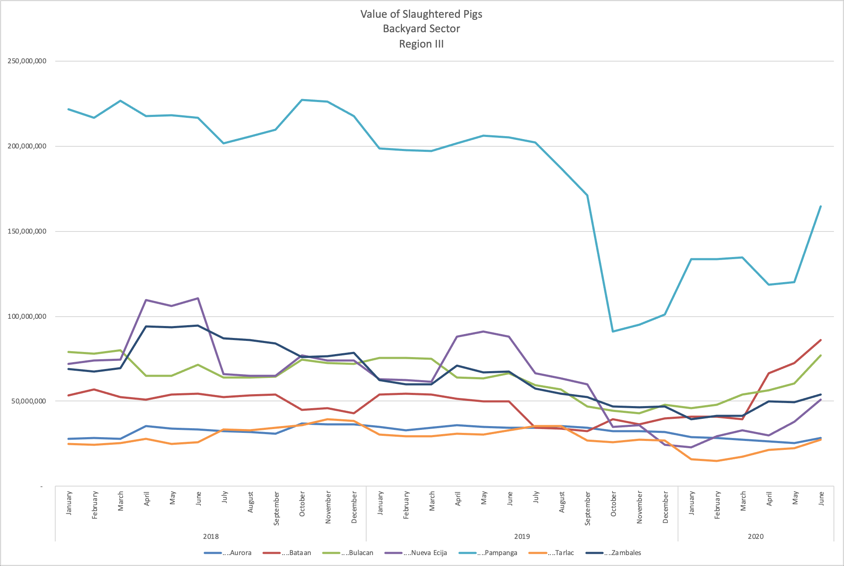


Figure 10: Estimated Total monthly value of pigs produced at backyard level slaughtered by province in Region III (Jan 2018 – June 2020)

Source: Farm Prices Survey (FPS) and Survey of Slaughterhouses & Poultry Dressing Plants of Philippine Statistics Authority (PSA).

This can be compared with the situation for commercial raising (Figure 11), where the value of hogs slaughtered in most provinces has remained relatively stable throughout 2018-2020, with the exception of Bulacan, where the value of hogs slaughtered from the commercial sector has had a major increase since October 2019. This increase in Bulacan is due to both an increase in the number of hogs slaughtered and also increases in the price due to the overall tightening of slaughter hog numbers in Region III. The increased slaughter numbers in the commercial sector in Bulacan over this period may be the major reason behind the decline in total swine population in the commercial sector in Region III over the same time period (see Figure 1).


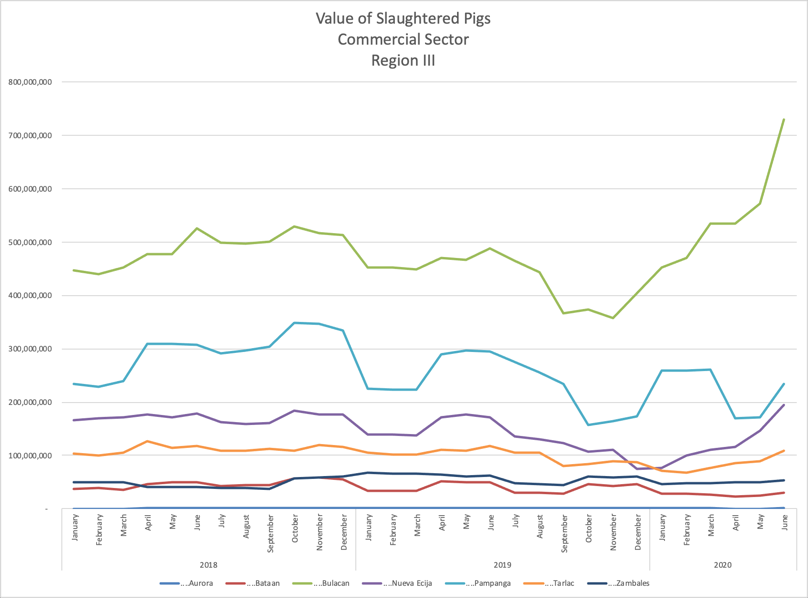


Figure 11: Estimated total monthly value of pigs produced at commercial level slaughtered by province in Region III (Jan 2018 – June 2020)

Source: Farm Prices Survey (FPS) and Survey of Slaughterhouses & Poultry Dressing Plants of Philippine Statistics Authority (PSA).

1. Survey of Slaughterhouses and Poultry Dressing Plants (SSHPDP) formerly known as Survey of Abattoir and Dressing Plants (SADP) (psa.gov.ph) [↑](#footnote-ref-1)
